# Bifurcate Regulation of Hematopoietic Homeostasis and Bone Osteogenesis by VHL‐HIF2α‐Controlled Adipocyte Function

**DOI:** 10.1002/advs.202509255

**Published:** 2025-11-08

**Authors:** Qian Li, Jia Li, Anshu Tang, Cong Li, Chao Zhang, Congcong Zhang, Yun‐Cai Liu

**Affiliations:** ^1^ Institute for Immunology and School of Basic Medical Sciences Tsinghua University Beijing 100084 China; ^2^ Tsinghua‐Peking Center for Life Sciences Tsinghua University Beijing 100084 China

**Keywords:** adipogenesis, osteosclerosis, hematopoiesis, VHL‐HIF axis

## Abstract

Adipocytes play a pivotal role in maintaining metabolic and immunological homeostasis. Here, this work shows that VHL‐HIF2α (VHL is Von Hippel‐Lindau) axis in mature adipocytes regulates hematopoiesis and osteogenesis. Genetic ablation of VHL in adipocytes triggers profound systemic autoinflammation and abnormal hematopoiesis, concomitant with fat mass decrease and pathological elevation of bone mass. On one hand, VHL deficiency results in aberrantly high stem cell factor (SCF) expression in adipocytes, which exerts a negative role for hematopoietic homeostasis through disrupting hematopoietic stem cell (HSC) quiescence. In vivo anti‐CD117 monoclonal antibody treatment ameliorates the hematopoietic defects in VHL‐deficient mice. On the other hand, direct HIF2α binding to hypoxia‐response elements in the *Rarres2* locus enhances chemerin production in adipocytes, which facilitates mesenchymal stem cell (MSC) osteogenesis via Wnt/β‐catenin activation. Pharmacological chemerin neutralization through CMKLR1 inhibition using α‐NETA mitigates osteogenic activity both in vitro and in vivo. This work thus identifies chemerin as the pivotal molecular nexus connecting hypoxic adipocyte dysfunction to pathological osteosclerosis. The findings uncover a hypoxia‐driven signaling network in adipocytes that orchestrates cross‐talk with both HSCs and MSCs to regulate systemic homeostasis, thereby revealing therapeutic targets for disorders associated with adipocyte dysfunction.

## Graphical abstract

The VHL‐HIF2α (VHL is Von Hippel‐Lindau) axis in adipocytes differentially regulates hematopoiesis and bone formation through stem cell factor (SCF) and chemerin, respectively. This hypoxia‐responsive pathway in adipocytes establishes a systemic signaling network with HSCs and MSCs to maintain tissue homeostasis, revealing a targetable axis for treating disorders linked to adipocyte dysfunction.

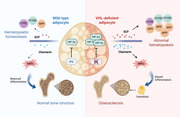

## 1 Introduction

Adipocytes are specialized cells that play essential roles in energy storage and endocrine signaling. Within adipose tissue (AT), adipocytes interact with stromal cells and immune cells to maintain metabolic and immunological homeostasis, through direct cell‐to‐cell contact as well as the secretion of pro‐ and anti‐inflammatory cytokines, chemokines, and metabolites.^[^ ^1^ ^]^

Bone marrow adipocytes (BMAs) originate from bone marrow mesenchymal stem cells (MSCs), which also differentiate into osteoblasts and chondroblasts.^[^ ^2^, ^3^ ^]^ Ectopic accumulation of BMAs is commonly associated with ageing and metabolic‐related disorders such as osteoporosis, dyslipidemia, and obesity.^[^ ^4^ ^]^ Most studies indicate that BMAs negatively regulate hematopoiesis and bone formation. Mice lacking BMAs exhibit enhanced expansion of hematopoietic progenitors and increased bone mass,^[^ ^5^, ^6^, ^7^ ^]^ while accumulation of BMAs suppresses the proliferation of hematopoietic progenitors and inhibits bone formation.^[^ ^8^, ^9^, ^10^, ^11^ ^]^ In contrast, BMAs have been reported to promote hematopoietic regeneration under certain conditions.^[^ ^12^, ^13^ ^]^

Hypoxia‐inducible factor‐alpha (HIFα) acts as a master regulator of cellular and systemic adaptations to hypoxia. Under normoxic conditions, HIFα undergoes hydroxylation mediated by prolyl hydroxylase domain (PHD) enzymes, thus recognized by the Von Hippel‐Lindau (VHL) E3 ubiquitin ligase complex, following ubiquitination and subsequent proteasomal degradation. In contrast, under hypoxic conditions, the enzymatic activity of PHDs is diminished, leading to the stabilization and accumulation of HIFα. Stabilized HIFα trans‐locates into the nucleus, where it forms a heterodimer with HIFβ and activates the transcription of hypoxia‐responsive genes.^[^ ^14^ ^]^ It has been well‐established that hypoxia signaling plays a pivotal role in modulating adipose tissue homeostasis and driving metabolic inflammation.^[^ ^15^ ^]^ Most of those studies have focused on the role of HIF‐mediated responses in the context of obesity, how VHL‐HIF axis in adipocytes is involved in regulating systemic homeostasis under steady state remains poorly understood.

Here, we generated adipocyte‐specific VHL knockout mice using the *Adipoq*‐Cre which targets BMAs and mature adipocytes in peripheral adipose tissues. VHL‐deficient mice exhibited systemic autoinflammation, abnormal hematopoiesis, fat mass decrease, and osteosclerosis. Genetic deletion of both *Vhl* and *Hif2a* completely rescued the phenotypic defects in VHL‐deficient mice. We next revealed that VHL‐HIF2α axis in adipocytes regulates lipid metabolism and adipogenesis, while controls hematopoietic homeostasis and bone formation in the bone marrow niche. VHL‐deficient adipocytes upregulate stem cell factor (SCF) expression, thus disturb HSC quiescence and promote HSC differentiation. We further identified that HIF2α drives the transcriptional activation of *Rarres2* (encoding chemerin) in adipocytes, which directly promotes bone formation through Wnt/β‐catenin signaling pathway. Our findings unveil how VHL‐HIF2α axis in mature adipocytes maintains bone microenvironment homeostasis. Understanding the mechanisms by which adipocytes regulate hematopoiesis and osteogenesis may pave the way for novel therapeutic strategies targeting diseases associated with adipocyte dysfunction.

## 2 Results

### 2.1 VHL Deficiency in Mature Adipocytes Leads to Systemic Autoinflammation

**Figure 1:**
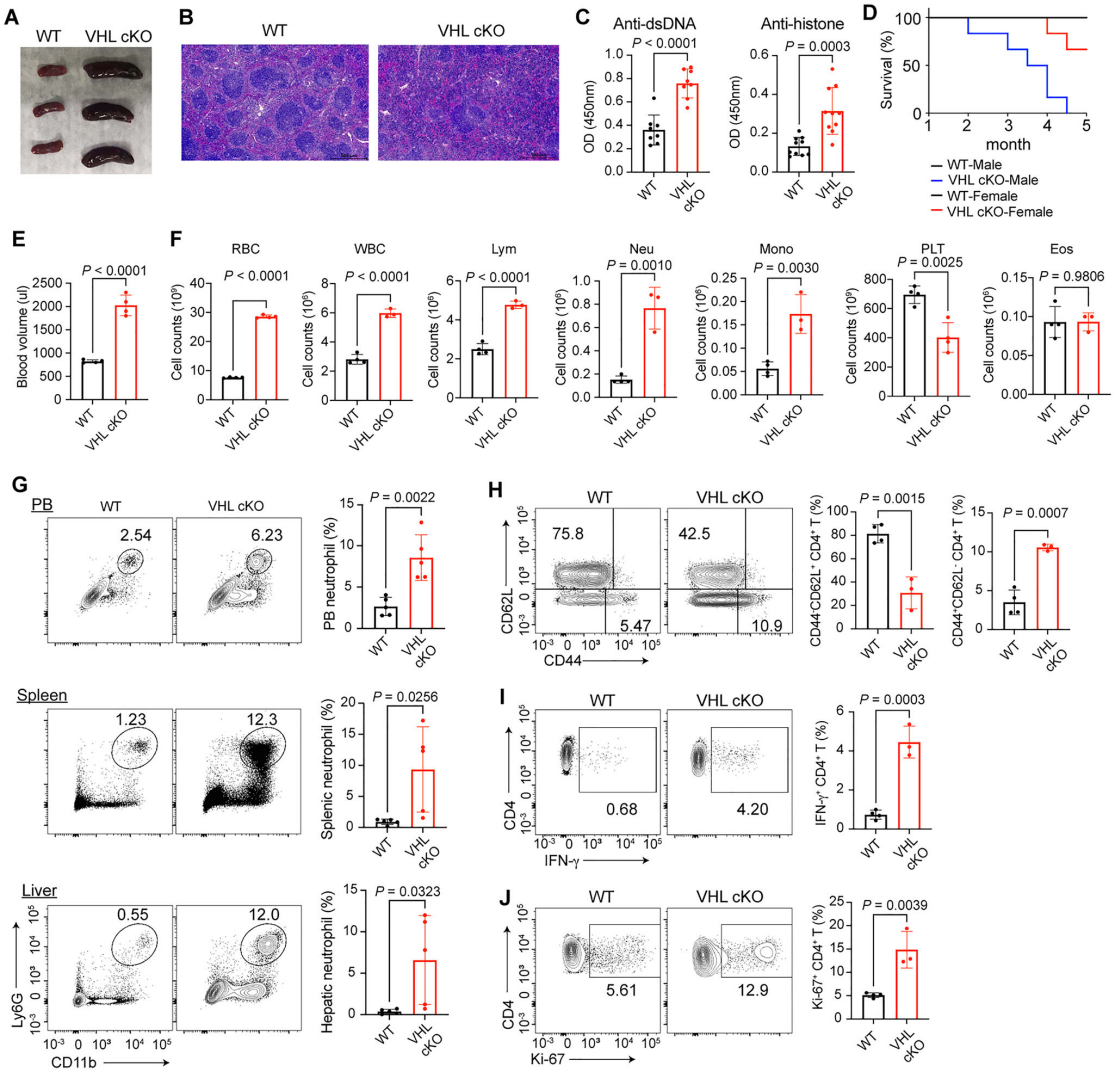
Von Hippel‐Lindau (VHL) deficiency in mature adipocytes results in systemic autoinflammation. A) Representative image of spleens from 8‐week‐old wild‐type (WT) and VHL cKO mice. B) H&E staining of spleen sections of 8‐week‐old WT and VHL cKO mice. Scale bars: 500 µm. C) ELISA of antibodies to double‐stranded DNA (anti‐dsDNA) and histone (anti‐histone) in the serum of 8‐week‐old male mice, presented as optical density at 450 nm (OD_450_). *n* = 8–10 mice per group. D) Survival rate of WT and VHL cKO mice. *n* = 6 mice per group. E) Peripheral blood (PB) volume of WT and VHL cKO mice. *n* = 4 mice per group. F) Total counts of red blood cells (RBC), white blood cells (WBC), lymphocytes (Lym), neutrophils (Neu), monocytes (Mono), platelets (PLT), and eosinophils (Eos) in the PB of WT and VHL cKO mice. *n* = 3–4 mice per group. G) Flow cytometric analysis and frequencies of neutrophils (CD45^+^ CD11b^+^ Ly‐6G^+^) from the PB, spleen and liver of WT and VHL cKO mice. Numbers indicate the frequencies of flow cytometric events. *n* = 5 mice per group. H) Flow cytometric analysis (left) and frequencies (right) of naive (CD44^–^CD62L^hi^) CD4^+^ T cells and effector‐like (CD44^hi^CD62L^−^) CD4^+^ T cells from PB of WT and VHL cKO mice. *n* = 3–4 mice per group. I,J), Flow cytometric analysis (left) and frequencies (right) of IFN‐γ^+^ (I) and Ki‐67^+^ (J) CD4^+^ T cells from PB of WT and VHL cKO mice. *n* = 3–4 mice per group. Data are represented as mean ± s.d. Statistical significance was assessed by two‐tailed unpaired Student's *t*‐test (C,E–J).

To investigate the role of VHL in mature adipocytes, we crossed *Vhl^fl/fl^* mice with *Adipoq‐*Cre mice to generate conditional VHL knockout mice (referred to as VHL cKO). We first identified that adipocyte‐specific genes, including *Adipoq, Lep*, *and Pparg*, are highly expressed in the adipose fraction (AF) compared to the stromal vascular fraction (SVF) of the epididymal white adipose tissue (eWAT) using reverse transcription PCR (RT‐PCR) (Figure S1A, Supporting Information). *Vhl* expression was depleted in AF of the VHL cKO mice, without affecting the non‐adipocytes in SVF (Figure S1B, Supporting Information). Remarkably, adult VHL cKO mice exhibited enlarged spleen with disrupted splenic architecture, represented by disorganized borders between white and red pulps (**Figure** **1**A,B).

VHL cKO mice showed elevated titers of autoantibodies to nuclear antigens such as double‐stranded DNA and histone IgG in the serum, indicative of autoinflammation (Figure 1C). Adipocyte‐specific deletion of VHL resulted in the death of adult mice starting at 2 months of age, with this phenomenon being more pronounced in male mice (Figure 1D). VHL cKO mice exhibited a significant increase in peripheral blood (PB) volume compared to wild‐type (WT) controls (Figure 1E). Hematological analysis revealed elevated counts of red blood cells (RBCs), white blood cells (WBCs), and major leukocyte subsets, including lymphocytes, neutrophils, and monocytes in PB of VHL cKO mice (Figure 1F). In contrast, platelet counts were markedly reduced in VHL cKO mice, while eosinophil levels remained comparable between the two groups (Figure 1F). Flow cytometric analysis revealed excessive neutrophil infiltration in multiple tissues of the VHL cKO mice, including the PB, spleen, and liver (Figure 1G). Additionally, CD4^+^ T cells and CD8^+^ T cells from the PB of VHL cKO mice exhibited an increased proportion of activated/memory‐like CD44^hi^CD62L^low^ populations and a reduced proportion of naive‐like CD44^low^CD62L^hi^ populations (Figure 1H, Figure S1C, Supporting Information). In addition, CD4^+^ T cells and CD8^+^ T cells from the PB of VHL cKO mice showed enhanced IFN‐γ production and proliferation, compared with those from WT mice (Figure 1I,J, Figure S1D,E, Supporting Information). Together, these data indicated that VHL deficiency in mature adipocytes plays a pivotal role in the development of systemic autoinflammation.

### 2.2 VHL Ablation in Adipocytes Attenuates Bone Marrow Hematopoiesis

**Figure 2:**
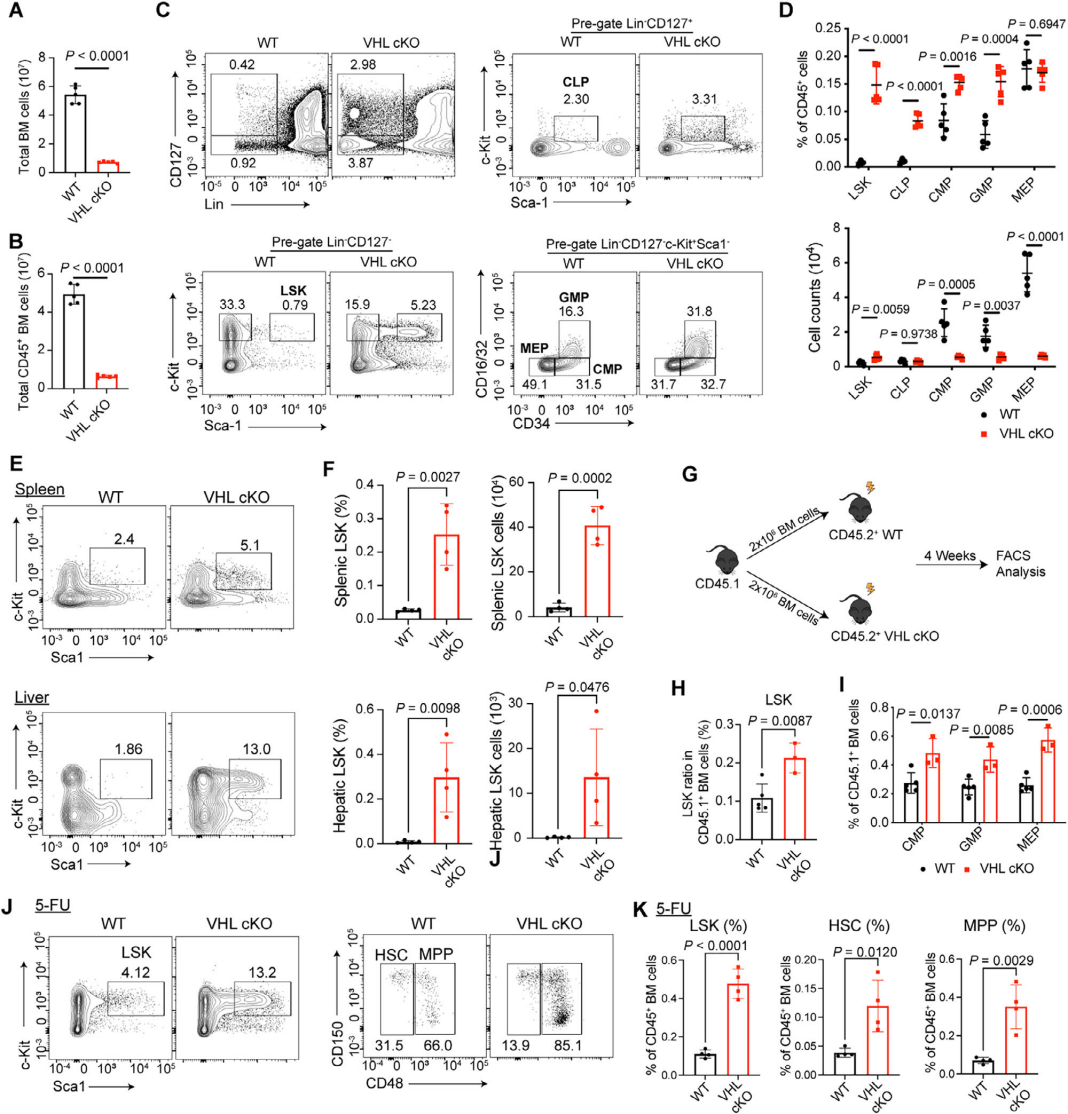
Von Hippel‐Lindau (VHL) ablation in adipocytes attenuates bone marrow hematopoiesis. A,B) Absolute numbers of total bone marrow cells (A) and CD45+ bone marrow cells (B) from 6‐week‐old wild‐type (WT) or VHL cKO mice. *n* = 5 mice per group. C) Gating strategy and flow cytometric analysis of hematopoietic progenitors from the bone marrow of 6‐week‐old WT and VHL cKO mice. *n* = 5 mice per group. BM progenitors were identified as LSK (Lin‐ CD127‐Sca‐1^+^c‐Kit^+^); CLP (Lin‐CD127^+^Sca‐1^lo^c‐Kit^lo^); CMP (Lin‐CD127‐Sca‐1‐c‐Kit^+^CD34^hi^CD16/32^lo^), GMP (Lin‐CD127‐Sca‐1‐c‐Kit^+^CD34^hi^CD16/32^hi^), and MEP (Lin‐CD127‐Sca‐1‐c‐Kit^+^CD34^lo^CD16/32^lo^). D) Frequencies (up) and absolute numbers (down) of LSKs, CLPs, CMPs, GMPs, and MEPs in (C). *n* = 5 mice per group. E) Flow cytometric analysis of LSKs in pre‐gated Lin‐CD127‐ cells from the spleen and liver of WT and VHL cKO mice. F) Frequencies (left) and absolute numbers (right) of LSKs in the spleen and liver of WT and VHL cKO mice. *n* = 4 mice per group. G) Schematic diagram of bone marrow regeneration. H) Frequencies of donor‐derived (CD45.1^+^) LSK cells in WT or VHL cKO recipients. *n* = 3–5 mice per group. I) Frequencies of donor‐derived (CD45.1^+^) CMP, GMP, MEP cells in WT or VHL cKO recipients. *n* = 3–5 mice per group. J) Gating strategy and flow cytometric analysis of LSK, HSC (Lin‐ CD127‐Sca‐1^+^c‐Kit^+^CD48^−^), and multipotent progenitor cell (MPP) (Lin‐ CD127‐Sca‐1^+^c‐Kit^+^CD48^+^) in the bone marrow collected 10 days after 5‐FU injection. K) Quantification of LSK, hematopoietic stem cell (HSC), and MPP frequencies in WT and VHL cKO mice after 5‐FU injection. *n* = 4 mice per group. Data are represented as mean ± s.d. Statistical significance was assessed by two‐tailed unpaired Student's *t*‐test (A,B,D,F,H,K) or multiple unpaired *t*‐tests (I).

Hematopoietic progenitors in the bone marrow are responsible for the production and replenishment of all immune cells, thereby sustaining the immune system. Recent studies have highlighted bone marrow adipocytes as a key regulator of hematopoietic homeostasis.^[^ ^2^ ^]^ Our analysis revealed a significant reduction in total bone marrow cellularity and CD45^+^ hematopoietic cells in adult VHL cKO mice compared to controls, suggesting impaired hematopoietic function (**Figure** **2**A,B). Notably, we observed a significant expansion in the relative proportions of hematopoietic progenitors in VHL cKO mice, including Lin^−^Sca1^+^c‐kit^+^ (LSK) cells, common lymphoid progenitors (CLPs), common myeloid progenitors (CMPs), and granulocyte‐macrophage progenitors (GMPs) (Figure 2C,D). While total LSK cell numbers increased in VHL cKO mice, the absolute counts of CMPs, GMPs, and megakaryocyte‐erythroid progenitors (MEPs) were all significantly decreased (Figure 2D).

Under hematopoietic stress conditions caused by infections, hematologic malignancies or bone disorders such as osteopetrosis when bone marrow function becomes compromised, hematopoietic progenitors may mobilize into peripheral circulation and subsequently colonize secondary organs, leading to extramedullary hematopoiesis (EMH).^[^ ^16^ ^]^ We detected significant accumulations of LSK cells in both splenic and hepatic tissues of VHL cKO mice, demonstrating established EMH activity (Figure 2E,F).

As VHL cKO mice showed extensive abnormalities in the whole body, we explored the original tissue where autoinflammation initiated. To figure out this question, we analyzed WT and VHL cKO mice at different ages after weaning. VHL cKO mice at 18 days of age displayed normal phenotypes across various tissues compared with littermate controls, including comparable bone marrow cellularity and LSK cell proportion (Figure S2A, Supporting Information). But as mice getting older, the bone marrow of VHL cKO mice started to show obvious defects. While 3‐week‐old cKO mice exhibited normal bone marrow cellularity compared to controls, flow cytometric analysis revealed a significant reduction in CD45^+^ hematopoietic cells, due to a decreased proportion of CD45^+^ cells within the total marrow population (Figure S2B, Supporting Information). Notably, we detected a significantly increased LSK cell proportion in VHL cKO marrow (Figure S2B, Supporting Information). However, 3‐week‐old VHL cKO mice displayed normal splenic size and architecture comparable to WT littermates (Figure S2C,D, Supporting Information). Furthermore, serum autoantibody titers showed no statistically significant differences, suggesting preserved immune homeostasis at this developmental stage (Figure 2E). Besides, the ratio of neutrophils in peripheral tissues were similar between 3‐week‐old VHL cKO and WT mice (data not shown).

We performed competitive bone marrow transplantation assays by mixing equal numbers of total bone marrow cells from CD45.2^+^ WT or VHL cKO donors with CD45.1^+^ WT competitor cells, followed by transplantation into lethally irradiated CD45.1^+^ recipients. Analysis was conducted 8 weeks post‐transplantation (Figure S2F, Supporting Information). We observed that when WT CD45.2⁺ donor cells were mixed with CD45.1⁺ competitor cells and transplanted into recipient mice, the chimerism of WT CD45.2⁺ cells in bone marrow (total CD45⁺, B220⁺ B cells, and LSK populations) and spleen (total CD45⁺ and B220⁺ B cells) remained approximately 50% (Figure S2G,H, Supporting Information). In contrast, VHL cKO CD45.2⁺ donor cells achieved significantly higher chimerism across all examined populations in both tissues, with a comparable magnitude of elevation observed among different cell types (Figure S2G,H, Supporting Information). The uniformly enhanced chimerism is likely attributable to the pre‐existing elevated frequency of LSK cells in zhe VHL cKO donor bone marrow.

Importantly, under competitive conditions after 8 weeks of regeneration, the ratios of hematopoietic progenitors (LSKs, CLPs, CMPs, GMPs, and MEPs) derived from CD45.2^+^ WT or VHL cKO donor cells were comparable within the total CD45.2^+^ bone marrow population (Figure S2I,J, Supporting Information). These results collectively indicate that the observed hematopoietic effects are mediated by niche‐dependent mechanisms rather than cell‐autonomous defects in hematopoietic progenitors.

In addition, CD45.2^+^ WT or VHL cKO mice at 4 weeks of age were lethally irradiated and subsequently transplanted with bone marrow cells isolated from CD45.1^+^ WT donors (Figure 2G). Four weeks after transplantation, flow cytometric analysis revealed a significant increase in the frequency of donor‐derived (CD45.1^+^) LSK cells in VHL cKO recipients compared to WT controls (Figure 2H). Similarly, the proportions of donor‐derived (CD45.1^+^) CMPs, GMPs, and MEPs were all elevated in VHL cKO recipients (Figure 2I). These results indicated that the VHL cKO microenvironment enhances the regenerative capacity of transplanted hematopoietic progenitors.

To assess the role of adipocyte VHL signaling in stress hematopoiesis, we challenged mice with 5‐fluorouracil (5‐FU) to induce myeloablation. Analysis 10 days post‐injection revealed significantly elevated proportions of LSK cells, hematopoietic stem cells (HSCs), and multipotent progenitor cells (MPPs) in the bone marrow of VHL‐deficient mice compared to WT controls (Figure 2J,K). These findings indicate that VHL deficiency in adipocytes enhances hematopoietic regeneration following chemotoxic stress. Taken together, our data demonstrated that VHL in adipocytes regulates bone marrow hematopoietic homeostasis and the occurrence of EMH in peripheral organs.

### 2.3 VHL‐Deficient Adipocytes Facilitate Osteogenesis

**Figure 3:**
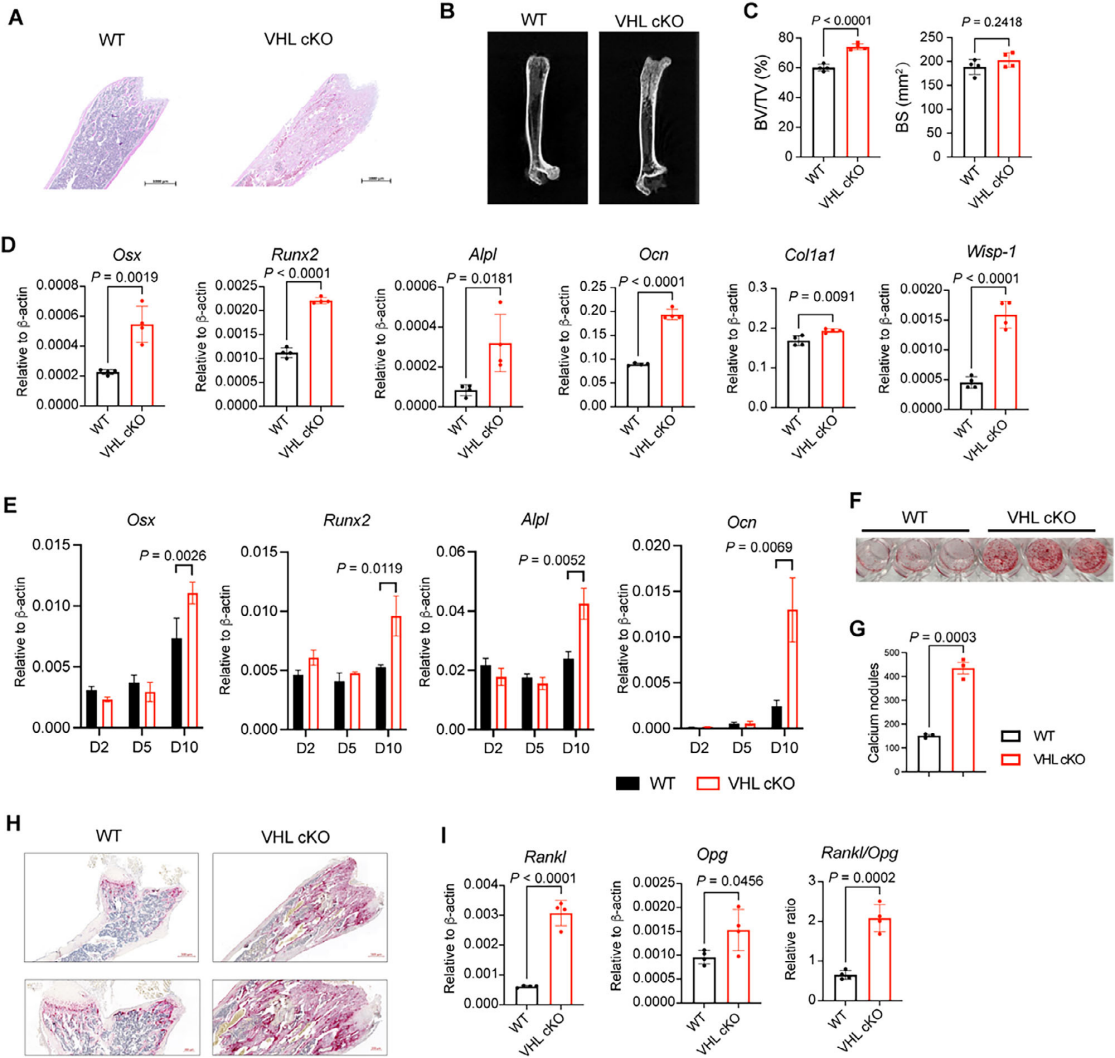
Von Hippel‐Lindau (VHL) deficient adipocytes facilitate osteosclerosis. A) Representative H&E staining of femur sections of 8‐week‐old wild‐type (WT) or VHL cKO mice. Scale bars: 1000 µm. B) Representative radiographs of femurs of 8‐week‐old WT and VHL cKO mice. C) The ratio of the segmented bone volume to the total volume volume (BV/TV, %) and the surface area (BS) of the femurs in (B). *n* = 4 mice per group. D) mRNA expression of *Osx, Runx2, Alpl, Ocn, Col1a1*, and *Wisp‐1* in total cells from the femur of 8‐week‐old WT and VHL cKO mice. E–G) Bone marrow mesenchymal stem cells (MSCs) from WT or VHL cKO mice were cultured under osteoblast‐induction conditions. E) mRNA expression of of *Osx, Runx2*, *Alpl*, and *Ocn* in BM‐MSC‐derived cells from WT or VHL cKO mice on day 2, day 5, and day 10 post induction. F) Alizarin Red S staining indicating osteogenic phenotype of bone marrow MSC‐derived cells from WT or VHL cKO mice under osteoblast‐induction conditions on day 14. The representative captured image of 48‐well plate was displayed. G) Numbers of calcium nodules in (F). H) Representative TRAP staining of femur sections of 8‐week‐old WT and VHL cKO mice. Scale bars: 500 µm (up); 200 µm (down). I) mRNA expression of of *Rankl*, *Opg*, and the relative expression ratio of *Rankl/Opg* in total cells from the femur of WT or VHL cKO mice. Data are represented as mean ± s.d. Statistical significance was assessed by two‐tailed unpaired Student's *t*‐test (C,D,G,I) or multiple unpaired *t*‐tests (E).

Histological analysis of the femurs showed remarkably increased total bone volume and trabecular bone volume in adult VHL cKO mice, responsible for decreased marrow cell deposition (**Figure** **3**A). Analysis of bone microarchitecture using microcomputed tomography (micro‐CT) revealed a significantly higher bone volume/total volume (BV/TV) ratio in adult VHL cKO mice compared with WT mice, whereas the bone surface area showed no significant difference (Figure 3B,C). In addition, VHL cKO mice showed elevated trabecular bone formation as young as 3 weeks of age (Figure S3A, Supporting Information). These data indicated that VHL deficiency in adipocytes results in osteosclerosis.

Healthy bone maintains a well‐organized balance between the osteoblast‐induced bone formation and osteoclast‐mediated bone resorption.^[^ ^17^ ^]^ Thus, we explored whether osteosclerosis in VHL cKO mice was due to osteoblast dysfunction. RT‐PCR analysis revealed that the total bone cells from VHL cKO mice showed significantly elevated expression of osteoblast‐specific genes, including runt‐related transcription factor‐2 (*Runx2*), osterix *(Osx)*, alkaline phosphatase *(Alpl)*, osteocalcin *(Ocn)*, collagen type I alpha (*Col1a1*), and wnt1‐inducible signaling pathway protein 1 (*Wisp‐1*), compared with that of WT mice (Figure 3D).

Osteoblasts originate from bone marrow MSCs which are also capable of giving rise to adipocytes and chondrocytes.^[^ ^2^ ^]^ We characterized bone marrow MSC cells as CD45^−^CD31^−^Sca1^+^CD29^+^CD24^+^ cells and adipocyte progenitors as CD45^−^CD31^−^Sca1^+^CD29^+^CD24^−^ cells (Figure S3B, Supporting Information), based on previous studies.^[^ ^18^, ^19^ ^]^ RT‐PCR analysis showed that adipocyte progenitors express high leptin, but low adiponectin, while mature adipocytes express high adiponectin (Figure S3C, Supporting Information). Bone marrow MSCs and adipocyte progenitors have comparable *Vhl* expression levels between WT and VHL cKO mice, confirming that VHL was specifically deleted in mature adipocytes, but not in progenitors (Figure S3D, Supporting Information). To investigate whether VHL deficiency in adipocytes affects bone marrow MSC osteogenesis, we established an in vitro bone marrow MSC‐derived osteoblast differentiation assay according to a previous report (Figure S3E, Supporting Information).^[^ ^20^ ^]^ Compared with MSCs from WT mice, we observed elevated expression of osteogenesis‐specific genes such as *Osx*, *Runx2*, *Ocn*, and *Alpl* in MSCs from VHL cKO mice under osteoblast differentiation conditions at 10 days post‐induction (Figure 3E). Additionally, MSCs derived from VHL cKO mice exhibited significantly enhanced osteoblastogenic potential, as demonstrated by increased bone mineralization staining with Alizarin Red S under osteoblast‐induction conditions (Figure 3F,G). These data confirmed that VHL deficiency in adipocytes promotes bone marrow MSC‐derived osteoblast differentiation.

Furthermore, we explored whether VHL‐deficient adipocytes directly affected MSC osteogenic differentiation using the bone marrow stromal cell line OP9. OP9 cells were first cultured for 3 days with fresh isolated mature adipocytes from the eWAT of WT or VHL cKO mice, followed by osteoblast‐induction for 14–21 days. The number of osteoblast colonies from OP9 cells incubated with VHL‐deficient adipocytes increased remarkably compared with the control group incubated with WT adipocytes, thus indicating a direct role of VHL‐deficient adipocytes on osteoblast differentiation (Figure S3F,G, Supporting Information).

We questioned whether osteoclastic bone resorption was responsible for osteosclerosis in VHL cKO mice. However, bone histomorphometric analysis of femurs using tartrate‐resistant acid phosphatase activity (TRAP) staining implied increased osteoclast activity in VHL cKO mice (Figure 3H). The receptor activator of nuclear factor‐κB ligand (RANKL)/RANK/ osteoprotegerin (OPG) signaling plays a key for osteoclast formation and bone resorption.^[^ ^21^ ^]^ We found that the expression of osteoclast‐specific gene *Rankl* was significantly upregulated in the bone of VHL KO mice, as well as the relative ratio of *Rankl*/*Opg*, suggesting elevated osteoclastogenesis (Figure 3I). These results indicated that the bone of VHL cKO mice exhibited increased osteoclast differentiation, which is generally negatively correlated with bone mass increase. Collectively, we concluded that VHL‐deficient adipocytes promote MSC osteoblastogenesis and thus result in osteosclerosis.

### 2.4 HIF2α Contributes to VHL‐Mediated Regulation of Adipocytes

**Figure 4:**
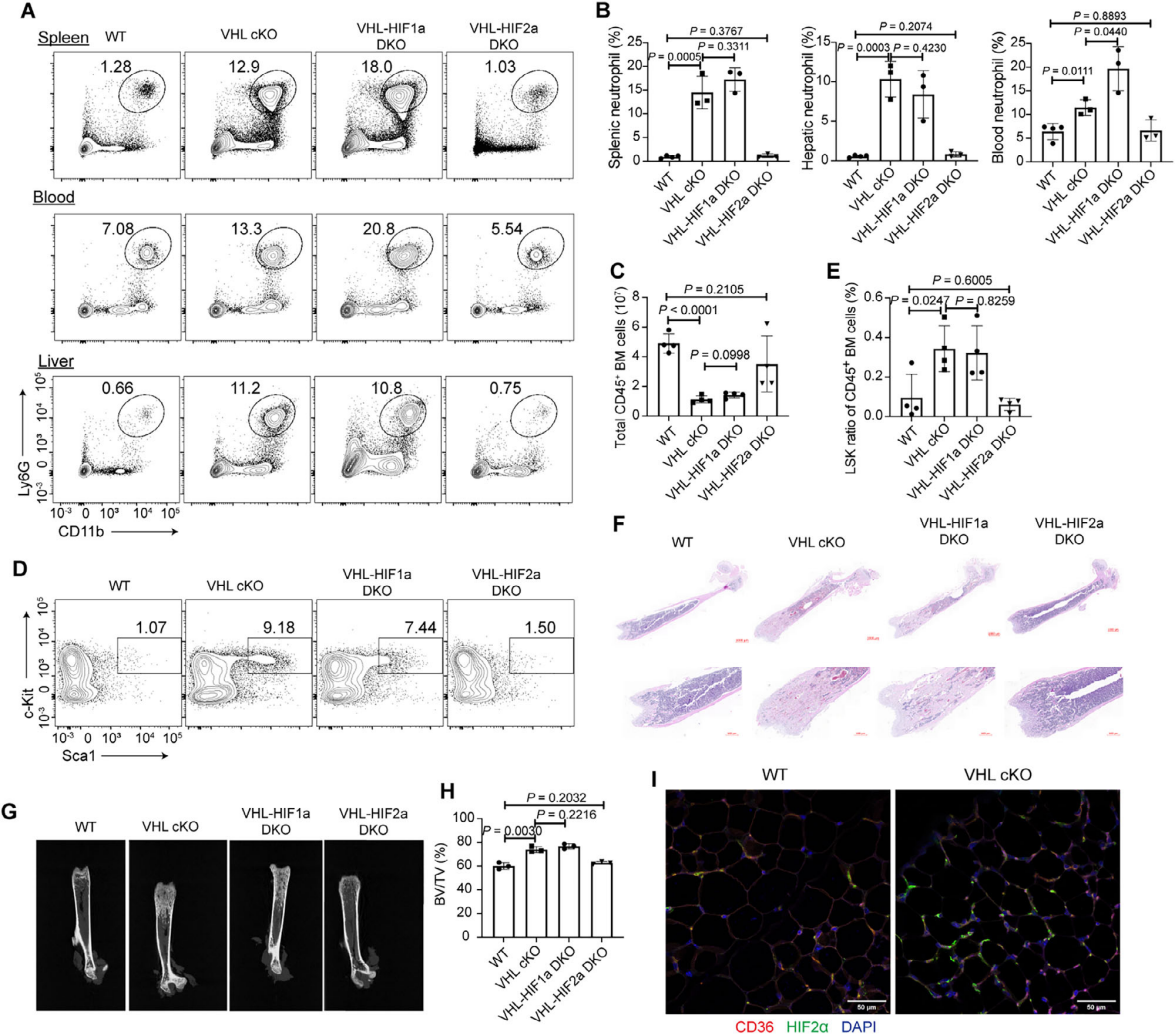
VHL‐HIF2α (VHL is Von Hippel‐Lindau) axis in adipocytes regulates systemic homeostasis. A) Flow cytometric analysis of neutrophils (CD45^+^ CD11b^+^ Ly‐6G^+^) from the spleen, blood, and liver of wild‐type (WT), VHL cKO, VHL‐HIF1α DKO, and VHL‐HIF2α DKO mice. B) Neutrophil frequencies from (A). *n* = 3–4 mice per group. C) Absolute numbers of CD45+ cells from the bone marrow of WT, VHL cKO, VHL‐HIF1α DKO, and VHL‐HIF2α DKO mice. *n* = 4 mice per group. D) Flow cytometric analysis of LSKs in pre‐gated Lin‐CD127‐ cells from the bone marrow of WT, VHL cKO, VHL‐HIF1α DKO, and VHL‐HIF2α DKO mice. E) Frequencies of bone marrow LSKs from WT, VHL cKO, VHL‐HIF1α DKO, and VHL‐HIF2α DKO mice. *n* = 4 mice per group. F) Representative H&E staining of femur sections of 8‐week‐old WT, VHL cKO, VHL‐HIF1α DKO, and VHL‐HIF2α DKO mice. Scale bars: 1000 µm (up); 500 µm (down). G) Representative radiographs of femurs of 8‐week‐old WT, VHL cKO, VHL‐HIF1α DKO, and VHL‐HIF2α DKO mice. H) BV/TV (%) of the femurs in (G). *n* = 4 mice per group. I) Representative sections of the EWAT from WT and VHL cKO mice stained for CD36 (red), HIF2a (Green), and DAPI (blue). Scale bars: 50 µm. Data are represented as mean ± s.d. Statistical significance was assessed by two‐tailed unpaired Student's *t*‐test (B,C,E,H).

It is well‐established that HIF1α and HIF2α are the major targets of VHL‐mediated ubiquitin‐dependent proteasomal degradation.^[^ ^14^ ^]^ To verify whether HIF1α or HIF2α is responsible for the defects in VHL cKO mice, we generated *Adipoq‐*Cre *Vhl^fl/fl^ Hif1a^fl/fl^* mice (VHL‐Hif1α DKO mice) and *Adipoq*‐Cre *Vhl^fl/fl^ Hif2a^fl/fl^* mice (VHL‐Hif2α DKO mice). We found that VHL‐Hif2α DKO mice reversed the appearance of splenomegaly observed in VHL‐deficient mice, whereas VHL‐HIF1α DKO mice failed (Figure S4A, Supporting Information). Flow cytometric analysis showed that adult VHL‐HIF2α DKO mice completely restored the abnormal neutrophil infiltration in the spleen, blood, and liver of VHL cKO mice (**Figure** **4**A,B).

We further analyzed the features of bone marrow hematopoiesis and bone structure in adult VHL‐Hif1α DKO and VHL‐Hif2α DKO mice. VHL‐Hif2α DKO mice completely restored the decreased numbers of CD45^+^ cells in the bone marrow of VHL cKO mice, while VHL‐HIF1α DKO mice failed (Figure 4C). In addition, VHL‐Hif2α double deficiency specifically rectified the elevated proportion of bone marrow LSK cells (Figure 4D,E). H&E staining and micro‐CT imaging analysis of bone microarchitecture revealed that the increased bone mass was completely restored in VHL‐Hif2α DKO mice, but not in VHL‐Hif1α DKO mice (Figure 4F–H). Immunofluorescence analysis revealed that HIF2α proteins accumulated in VHL‐deficient adipocytes (Figure 4I).

Besides, VHL‐Hif2α DKO mice rescued the abnormal EMH in the periphery, as the LSK ratios and numbers in the spleen and liver all dropped back (Figure S4B, S4C, Supporting Information). Additionally, in the in vitro osteoblast differentiation assay, bone marrow MSCs from VHL‐HIF2α DKO mice reversed the elevated osteoblast differentiation potential in VHL cKO mice, whereas MSCs from VHL‐HIF1α DKO mice did not reverse the changes (Figure S4D, Supporting Information). Collectively, these data demonstrated that HIF2α but not HIF1α plays an essential role in VHL‐manipulated regulation of adipocytes.

### 2.5 VHL Ablation in Adipocytes Impairs Lipid Metabolism and Results in Fat Mass Decrease

**Figure 5:**
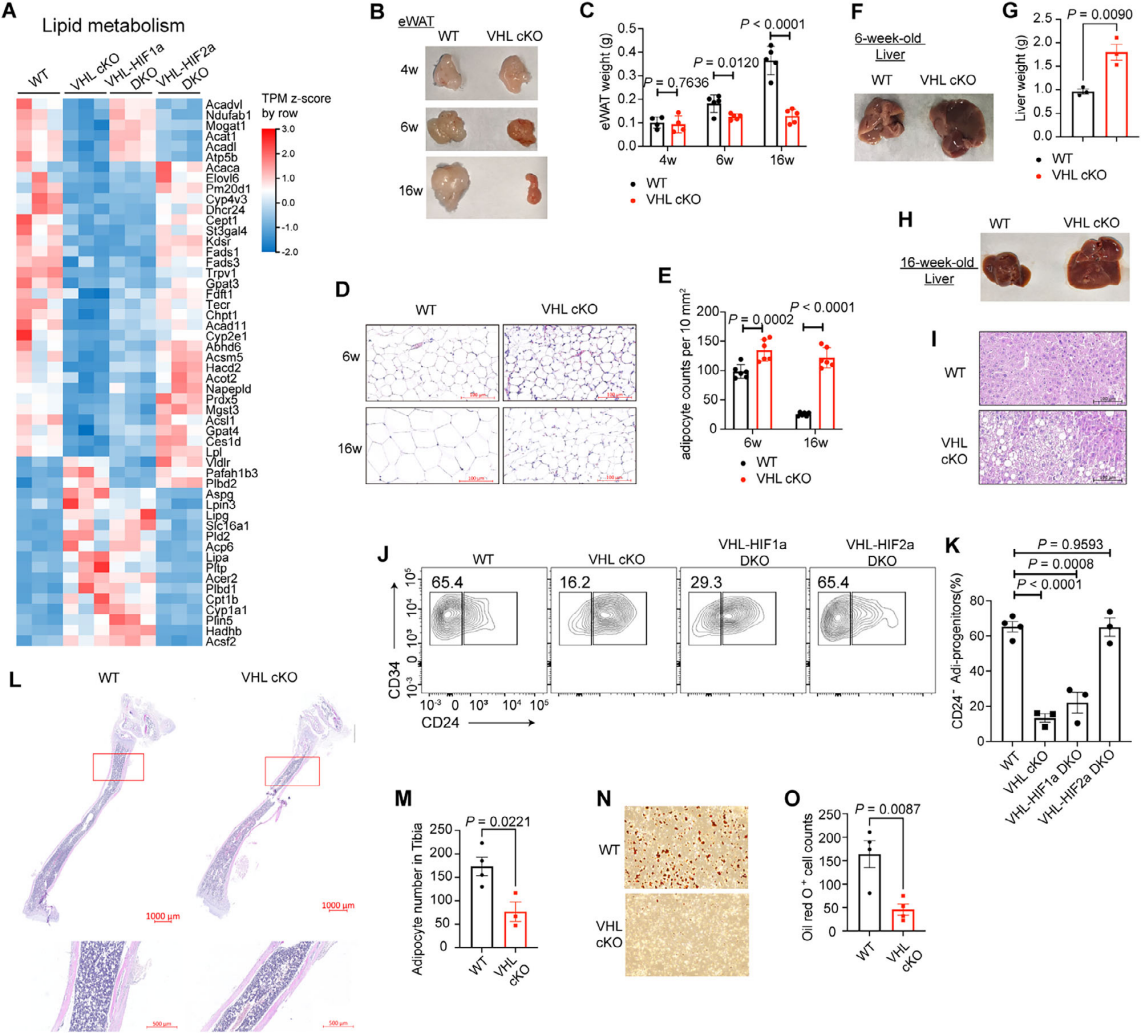
Von Hippel‐Lindau (VHL) ablation in adipocytes impairs lipid metabolism and results in adipocyte dysfunction. A) Heatmap of lipid metabolism‐related genes in adipocytes of wild‐type (WT), VHL cKO, VHL‐HIF1α DKO, and VHL‐HIF2α DKO mice. B) Representative image of eWAT from WT and VHL cKO mice at 4, 6, or 16 weeks of age. C) Weights of eWAT in WT and VHL cKO mice at 4, 6, or 16 weeks of age. *n* = 3–5 mice per group. D) Representative H&E staining of eWAT sections of WT or VHL cKO mice at 6 or 16 weeks of age. Scale bars: 100 µm. E) Statistical counts of adipocytes in 10 mm^2^ eWAT of WT and VHL cKO mice at 6 or 16 weeks of age. *n* = 6 views per group. F) Representative image of liver from 6‐week‐old WT and VHL cKO mice. G) Weights of liver in 6‐week‐old WT and VHL cKO mice. *n* = 3 mice per group. H) Representative image of liver from 16‐week‐old WT and VHL cKO mice. I) Representative H&E staining of liver sections of 16‐week‐old WT or VHL cKO mice. Scale bars: 100 µm. J) Flow cytometric analysis of CD24‐ adipocyte progenitors in pre‐gated CD45^−^CD31^−^Sca1^+^CD29^+^ cells in the bone marrow of WT, VHL cKO, VHL‐HIF1α DKO, and VHL‐HIF2α DKO mice. K) Frequencies CD24‐ adipocyte progenitors in (J). *n* = 3–4 mice per group. L) H&E staining of bone marrow space in tibia of 4‐week‐old WT and VHL cKO mice. Boxed areas in distal tibia show high magnification. Scale bars: 1000 µm (up); 500 µm (down). M) Total counts of mature adipocytes in distal tibia (L). *n* = 3–4 mice per group. N) Representative Oil Red O staining of bone marrow mesenchymal stem cells (MSCs) from WT or VHL cKO mice under adipocyte‐induction conditions. O) Statistical counts of Oil Red O+ adipocytes in (N). Data are represented as mean ± s.d. Statistical significance was assessed by two‐tailed unpaired Student's t‐test (G,K,M,O) or multiple unpaired t‐tests (C,E).

Although HIF1α and HIF2α share structural similarities, they have distinct roles in regulating metabolic processes. We performed bulk RNA‐seq and compared the gene expression patterns of mature adipocytes from the eWAT of wild‐type, VHL cKO, VHL‐Hif1α DKO, and VHL‐Hif2α DKO mice. Transcriptional profile analysis of VHL cKO versus WT adipocytes identified 2706 genes that were significantly changed, with 1328 genes upregulated and 1378 genes downregulated (data not shown). Gene expression analysis showed that VHL‐Hif1α DKO adipocytes restored the expression of most genes involved in glycolysis/glucogenesis, TCA cycle, and oxidative phosphorylation, while lipid metabolism‐associated genes could be restored mainly by VHL‐HIF2α double deficiency, indicating that HIF1α and HIF2α respectively regulates the glucose and lipid metabolism of adipocytes (**Figures** **5**A and S5A, Supporting Information). In detail, we found that HIF2α negatively regulates the expression of genes related to lipid synthesis, such as *Acaca*, *Elovl6*, *Fads1*, *Fads3*, *Gpat3*, *Gpat4*, and *Acsl1*, while positively regulates the genes related to lipolysis or fatty acid oxidation, such as *Lipa*, *Plin5*, *Cpt1b*, and *Acsf2* (Figure 5A).

Although 4‐week‐old VHL cKO mice showed normal eWAT pad sizes compared with WT controls, adult VHL cKO mice developed a pronounced reduction in eWAT mass (Figure 5B,C). This phenotypic disparity progressively exacerbated as mice aged (Figure 5B,C). Histological analysis revealed a marked reduction in adipocyte size within the eWAT of adult VHL cKO mice compared to littermate controls, consistent with the observed decline in fat mass (Figure 5D,E). We also observed decreased size of inguinal WAT (iWAT) and brown adipose tissue (BAT) in adult VHL cKO mice (Figure S5B,C, Supporting Information). Targeted lipidomics analysis showed that most lipid species exhibited a decreased tendency in VHL‐deficient mature adipocytes, especially PC (Phosphatidylcholine), PE (Phosphatidylethanolamine), and PI (Phosphatidylinositol) (Figure S5D, Supporting Information). Oppositely, lipid contents in the serum of VHL cKO mice showed an increased tendency, especially Cer (Ceramide), PC, PE, PG (phosphatidylglycerol), PI, and FA (fatty acid) (Figure S5E, Supporting Information). Additionally, adult VHL cKO mice exhibited obvious hepatic hyperplasia (Figure 5F,G). We also observed spontaneous hepatic steatosis in 16‐week‐old VHL cKO mice, with obvious ectopic lipid droplets deposition in hepatic cells (Figure 5H,I).

To determine whether HIF isoforms exert conserved functions in bone marrow adipocytes under VHL deficiency, we isolated primary bone marrow adipocytes from the long bones of wild‐type, VHL cKO, VHL‐Hif1α DKO, and VHL‐Hif2α DKO mice and profiled the expression of key metabolic genes. RT‐PCR analysis confirmed efficient *Vhl* depletion in bone marrow adipocytes across all knockout genotypes. We found that HIF2α predominantly regulates the expression of *Acsl1*, a central gene in lipid synthesis, while HIF1α governs the expression of glycolysis‐associated *Pkm2* and TCA cycle‐related *Mdh2* (Figure S5F, Supporting Information). These results align with RNA‐seq data from white adipose tissue adipocytes, highlighting conserved roles of the VHL–HIF signaling axis across distinct adipose depots.

Flow cytometry analysis revealed that, compared with WT controls, the adipocytic differentiation potential of bone marrow MSC cells decreased in VHL cKO and VHL‐Hif1α DKO mice, but not in VHL‐Hif2α DKO mice, indicated by the ratio of CD24^−^ adipocyte progenitors (Figure 5J,K). H&E staining showed that the marrow cavity of the distal tibia in VHL cKO mice had less mature adipocytes compared with littermate controls (Figure 5L,M). We established an in vitro system for adipogenic differentiation of bone marrow‐derived MSCs (Figure S5G, Supporting Information). The number of bone marrow MSC‐derived adipocytes from VHL cKO mice sharply decreased, compared with that from WT mice (Figure 5N,O).

We utilized OP9 cells to generate VHL‐deficient adipocytes through a retrovirus‐mediated shRNA knockdown system, followed by adipogenic differentiation assay (Figure S5H, Supporting Information). We observed a significant downregulation of *Vhl* gene expression in OP9 cells following shVhl‐mediated knockdown, regardless of adipogenic induction (Figure S5I, Supporting Information). VHL knockdown in OP9‐derived adipocytes (OP9‐Adi) resulted in decreased lipid droplet accumulation, indicating impaired adipogenesis (Figure S5J,K, Supporting Information). Taken together, we demonstrated that VHL‐Hif2α axis in adipocytes plays a critical role in regulating lipid metabolism and adipogenesis.

### 2.6 VHL‐Deficient Adipocytes Upregulate SCF Expression and Disturb HSC Quiescence

Bone marrow adipocytes play a complex and dynamic role in regulating hematopoiesis. Given that VHL cKO mice exhibited severe bone marrow hematopoietic defects, we explored whether VHL‐deficient adipocytes directly affect the quiescence and differentiation of hematopoietic progenitors.

**Figure 6:**
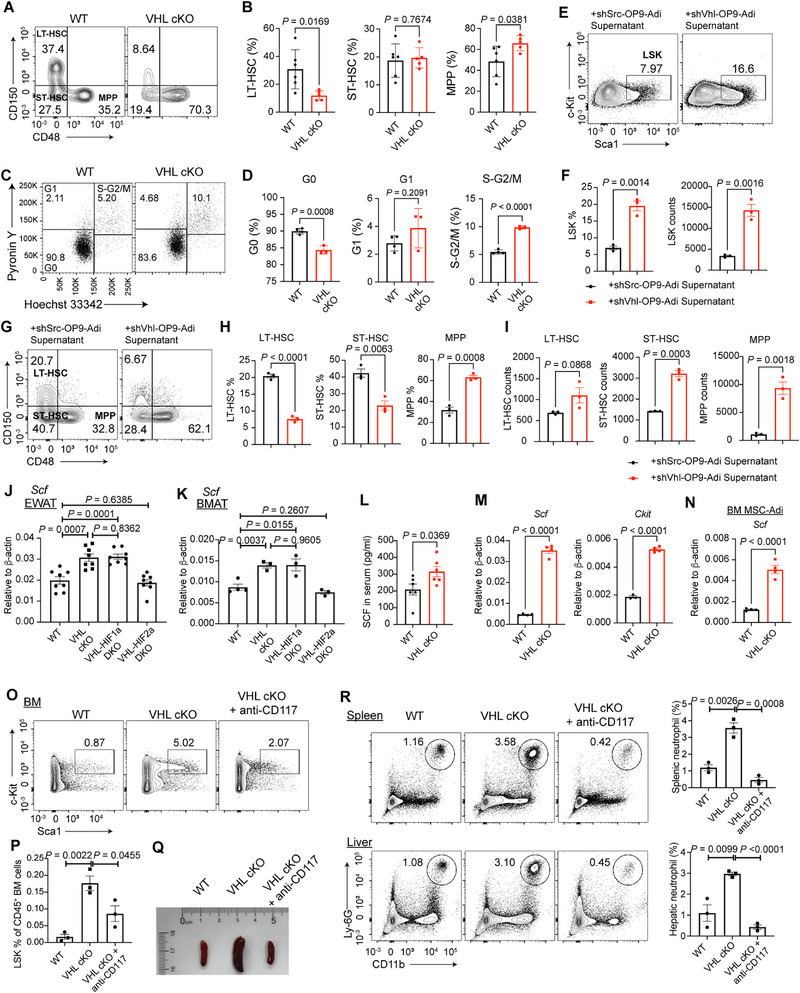
Von Hippel‐Lindau (VHL) deficient adipocytes up‐regulate SCF expression and disturb HSC quiescence. A) Flow cytometric analysis of long‐term hematopoietic stem cells (LT‐HSCs) (Lin‐CD127‐Sca‐1^+^c‐Kit^+^CD150^+^CD48^−^), short‐term HSCs (ST‐HSCs) (Lin‐CD127‐Sca‐1^+^c‐Kit^+^CD150^−^CD48^−^), and multipotent progenitor cells (MPPs) (Lin‐CD127‐Sca‐1^+^c‐Kit^+^CD150^−^CD48^+^) from pre‐gated LSKs of 6‐week‐old wild‐type (WT) and VHL cKO mice. B) Frequencies of LT‐HSCs, ST‐HSCs, and MPPs in LSKs from (A). *n* = 5–6 mice per group. C) Cell cycle analysis of LSKs from BM of 3‐week‐old mice through staining of Hoechst 33342 and Pyronin Y. D) Frequencies of BM LSKs at different cell cycle stages from WT or VHL cKO mice. *n* = 3–4 mice per group. E–I) FACS sorted LSKs were cultured with supernatants from shScramble (shSrc) or shVhl‐targeting OP9‐derived adipocytes (OP9‐Adi) and analyzed on day 3. E) Flow cytometric analysis of LSKs. F) Frequencies and numbers of LSKs in (E). G) Flow cytometric analysis of LT‐HSCs, ST‐HSCs, and MPPs from pre‐gated LSKs in (E). H and I) Frequencies (H) and numbers (I) of LT‐HSCs, ST‐HSCs, and MPPs in (G). J) mRNA expression of *Scf* in mature adipocytes from eWAT of WT, VHL cKO, VHL‐HIF1α DKO, and VHL‐HIF2α DKO mice. *n* = 2 mice (4 technical replicates each). K) mRNA expression of *Scf* in mature adipocytes isolated from bone marrow of WT, VHL cKO, VHL‐HIF1α DKO, and VHL‐HIF2α DKO mice. *n* = 3–4 technical replicates. L) Concentrations of SCF in the serum from WT and VHL cKO mice. *n* = 6 mice per group. M) mRNA expression of *Scf and Ckit* in total cells from the tibia of WT and VHL cKO mice. N) mRNA expression of *Scf* in bone marrow mesenchymal stem cell (MSC) derived adipocytes (BM MSC‐Adi) from WT and VHL cKO mice. O–R) VHL cKO mice were treated with anti‐CD117 monoclonal antibody (200 µg, i.p.) every other day for 2 weeks starting at 3 weeks of age, followed by analysis. O) Flow cytometric analysis of LSK cells in the bone marrow. P) Quantification of LSK population frequencies across indicated genotypes and treatments. *n* = 3 mice per group. Q) Representative image of spleens. R) Flow cytometric analysis and frequencies of neutrophils in the spleen and liver in indicated mice. *n* = 3 mice per group. Data are represented as mean ± s.d. Statistical significance was assessed by two‐tailed unpaired Student's *t*‐test (B,D,F,H–N,P,R).

Compared with WT control, the LSK population from the bone marrow of VHL cKO mice exhibited decreased portion of long‐term HSCs (LT‐HSCs) with reduced portion of multipotent progenitors (MPPs), while the ratio of short‐term HSCs (ST‐HSCs) did not significantly changed (**Figure** **6**A,B). As the proportion of LSKs in total CD45^+^ bone marrow cells increased in VHL cKO mice (Figure 2C, Figure S2B, Supporting Information), we evaluated the cell cycle status of LSKs by intracellular Hoechst 33342/Pyronin Y staining. We found that LSKs from the bone marrow of VHL cKO mice exhibited less quiescent G0 population but increased proliferating S‐G2/M population, compared with littermate controls (Figure 6C,D). Consistently, we observed significantly higher percentages of LSKs incorporated 5‐bromo‐2’‐deoxyuridine (BrdU) in the bone marrow and spleen of VHL cKO mice compared with control mice (Figure S6A,B, Supporting Information). Therefore, we speculated that the hematopoietic progenitors in the bone marrow niche of VHL cKO mice are less quiescent and have relatively higher cell cycle activity at steady state.

To testify whether VHL‐deficient adipocyte promote LSK differentiation through secreted factors, we used the OP9‐Adi assay. When cultured with the supernatants from VHL knockdown OP9‐Adi, the ratio and number of LSKs significantly increased compared with the control group (Figure 6E,F). In detail, LSK cells treated with the supernatants of VHL‐deficient OP9‐Adi exhibited decreased portion of LT‐HSCs and ST‐HSCs, along with increased ratio and number of MPPs, indicating loss of quiescence and elevated differentiation (Figure 6G–I). Besides, VHL knockdown OP9‐Adi boosted LSK proliferation in vitro, implied by Ki‐67 staining (Figure S6C,D, Supporting Information).

Adipocyte‐secreted factors play an important role in hematopoiesis, such as adiponectin, leptin, SCF, and CXCL12.^[^ ^2^, ^12^, ^22^ ^]^ SCF and its receptor c‐KIT play an essential role in the survival, migration, and differentiation of multiple hematopoietic stem and progenitor cells.^[^ ^23^ ^]^ Recent studies demonstrated that adipocyte‐derived SCF critically supports hematopoiesis under both physiological and metabolically stressed conditions.^[^ ^12^, ^24^ ^]^ Previous studies have identified that HIF2α directly induces SCF secretion in tumor cells through transcriptional activation.^[^ ^25^, ^26^ ^]^ RT‐PCR analysis revealed elevated expression levels of *Scf* in mature adipocytes from the eWATs and bone marrow of VHL cKO and VHL‐HIF1α dKO mice, which was rectified in that of VHL‐HIF2α dKO mice (Figure 6J,K). We also detected elevated concentration of SCF in the serum of VHL cKO mice compared with that of WT mice (Figure 6L). Additionally, we detected significantly increased expression of *Scf* and *Ckit* in the tibia of VHL cKO mice, indicating active SCF/c‐Kit signaling in the bone morrow niche (Figure 6M). Besides, bone marrow‐derived MSCs of VHL cKO mice under adipocyte‐induction conditions exhibited higher *Scf* expression, compared with those of WT controls (Figure 6N).

We performed in vivo anti‐CD117 monoclonal antibody treatment to determine whether direct blockade of SCF/c‐Kit signaling could rescue the hematopoietic defects in VHL cKO mice. Our results showed that anti‐CD117 treatment effectively mitigated the expansion of LSK cells in the bone marrow (Figure 6O,P) and ameliorated the splenomegaly induced by VHL deficiency (Figure 6Q). Furthermore, anti‐CD117 administration attenuated neutrophil infiltration in peripheral organs, including the spleen and liver (Figure 6R).

Taken together, these data indicated that VHL‐deficient adipocytes upregulate SCF expression, which disturbs HSC quiescence and promotes the proliferation and differentiation of hematopoietic progenitors, contributing to abnormal hematopoiesis.

### 2.7 VHL Deletion Drives Chemerin Expression in Adipocytes Through HIF2α

We testified whether the elevated bone marrow MSC osteogenesis efficacy in VHL cKO mice was caused by adipocyte‐secreted factors. WT bone marrow MSCs were cultured in osteoblast‐induction medium and supplemented with the supernatants collected from MSCs or MSC‐derived adipocytes (MSC‐Adi) (Figure S7A, Supporting Information). Compared with supernatants from MSCs cultured with common medium, supernatants from MSC‐Adi of WT mice evidently hindered osteogenesis (Figure S7B,C, Supporting Information). This was consistent with the common knowledge that adipocytes inhibit osteoblast differentiation. However, supernatants from MSC‐Adi of VHL cKO mice significantly promoted osteogenesis compared with that of WT mice, indicating that VHL‐deficient adipocytes directly enhance MSC osteoblastogenesis through secreted factors (Figure S7D,E, Supporting Information).

**Figure 7:**
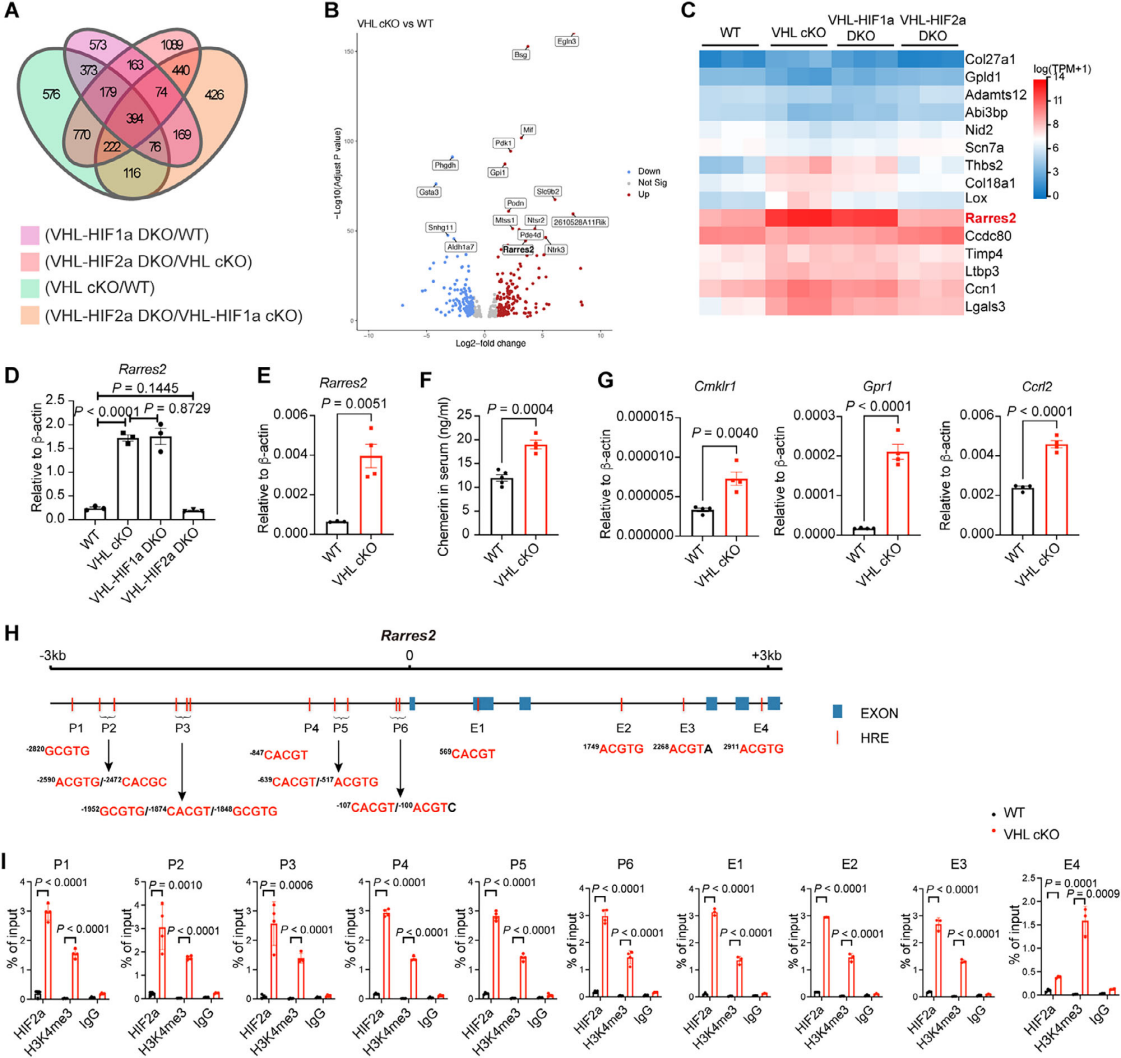
Von Hippel‐Lindau (VHL) deletion drives chemerin expression through HIF2α. A) Venn diagram showing the numbers of unique and overlapping genes changed between different groups. B) Volcano plot comparison of the 394 differentially regulated genes in the intersection in (A). C) Heatmap of 15 extracellular matrix proteins selected from the 394 genes. D) mRNA expression of *Rarres2* in mature adipocytes from eWAT of wild‐type (WT), VHL cKO, VHL‐HIF1α DKO, and VHL‐HIF2α DKO mice. E) mRNA expression of *Rarres2* in total cells from the tibia of WT and VHL cKO mice. F) Concentrations of chemerin in the serum from WT and VHL cKO mice. *n* = 4 mice per group. G) mRNA expression of *Cmklr1*, *Gpr1*, and *Ccrl2* in total cells from the tibia of WT and VHL cKO mice. H) Structural schematic of the mouse *Rarres2* gene with putative HREs. Blue boxes for exon and red boxes for HRE are shown. I) ChIP‐qPCR analysis of the mouse *Rarres2* gene loci with anti‐HIF2α, anti‐H3K4me3, and isotype control antibody (IgG) in eWAT mesenchymal stem cell (MSC) derived adipocytes from WT or VHL cKO mice. Results were normalized to input DNA. Data are represented as mean ± s.d. Statistical significance was assessed by two‐tailed unpaired Student's *t*‐test (D–G) or multiple unpaired *t*‐tests (I).

Via Venn diagram analysis, we found 394 genes specifically regulated by HIF2α in VHL‐deficient adipocytes, including 15 genes encoding extracellular matrix proteins (**Figure** **7**A,B). Of the genes encoding secreted proteins in VHL‐deficient adipocytes, *Rarres2*, which encodes the adipokine chemerin, was identified as a potential candidate (Figure 7B,C). The tremendously elevated expression levels of *Rarres2* in mature adipocytes from the eWATs of VHL cKO and VHL‐HIF1α dKO mice were verified by RT‐PCR (Figure 7D). Additionally, we detected significantly increased expression of *Rarres2* in the tibia of VHL cKO mice (Figure 7E). Also, bone marrow‐derived MSCs of VHL cKO mice under adipocyte‐induction conditions exhibited higher *Rarres2* expression, compared with those of WT controls (Figure S7F, Supporting Information). We also detected significantly enhanced concentration of chemerin in the serum of VHL cKO mice compared with that of WT mice (Figure 7F). Accordingly, the expression levels of chemerin receptors *Cmklr1*, *Gpr1*, and *Ccrl2* were notably enhanced in the bone and bone marrow‐derived MSC of VHL cKO mice under osteoblast‐induction conditions, compared with those of WT controls (Figure 7G and Figure S7G, Supporting Information).

To further understand the mechanisms by which HIF2α regulates *Rarres2* expression, we examined whether the *Rarres2* gene could serve as a direct target of HIF2α in adipocytes. Analysis of the *Rarres2* gene revealed that there are several putative HRE regions in this genetic locus (Figure 7H). A chromatin immunoprecipitation (ChIP) assay was performed to determine whether HIF2α interacts with those putative binding sites. Notably, we found that HIF2α associated with all of the identified HRE sites in MSC‐derived adipocytes (Figure 7I). Importantly, the HIF2α binding sites were correlated with the modification of histone H3 tri‐methylation at the lysine 4 residue (H3K4me3) in VHL‐deficient cells compared with WT controls (Figure 7I). These data suggested that HIF2α transcriptionally activate *Rarres2* expression.

### 2.8 Chemerin Promotes Osteogenesis Through Wnt/β‐catenin Signaling Pathway

Chemerin is an adipocyte‐derived adipokine highly expressed in adipose tissue, lung, and liver, which is commonly involved in metabolic and inflammatory pathologies.^[^ ^27^ ^]^ Recent studies have established high chemerin levels as a risk factor of osteoporosis.^[^ ^28^ ^]^

**Figure 8:**
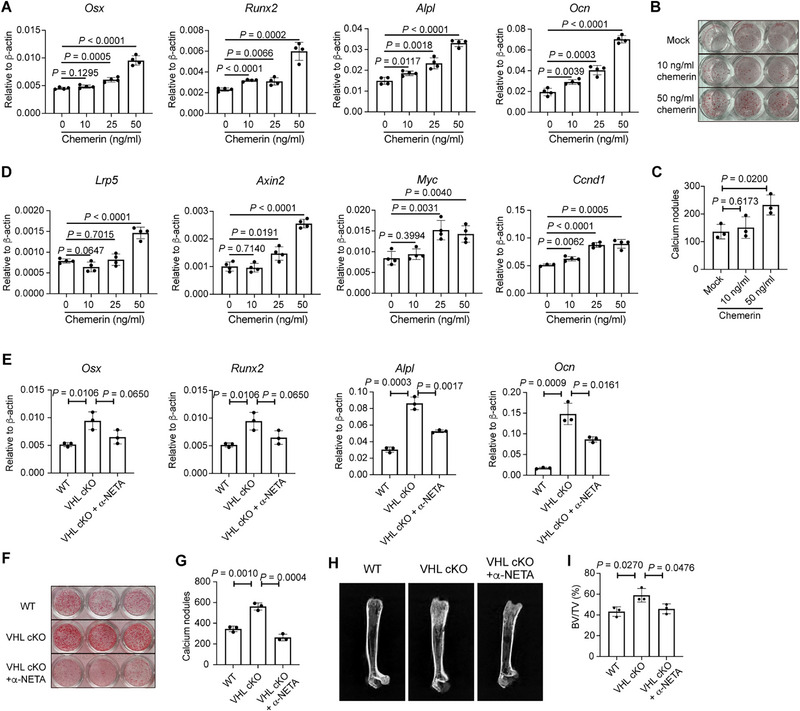
Chemerin promotes bone marrow mesenchymal stem cell (MSC) osteoblastogenesis. A) mRNA expression of *Osx*, *Runx2*, *Alpl*, and *Ocn* in bone marrow MSC‐derived osteoblasts on day 10 under osteoblast‐induction conditions with chemerin treatment. B) Alizarin Red S staining of MSC‐derived osteoblasts treated with chemerin. The representative captured image of 48‐well plate was displayed. C) Numbers of calcium nodules from (B). D) mRNA expression of *Lrp5*, *Axin2*, *Myc*, and *Ccnd1* in bone marrow MSC‐derived osteoblasts on day 10 under osteoblast‐induction conditions with chemerin treatment. E) mRNA expression of *Osx*, *Runx2*, *Alpl*, and *Ocn* in bone marrow MSC‐derived cells from wild‐type (WT) or Von Hippel‐Lindau (VHL) cKO mice on day 10 under osteoblast‐induction conditions with/without 20 × 10^−6^ m α‐NETA treatment. F) Alizarin Red S staining of MSC‐derived osteoblasts treated with α‐NETA. The representative captured image of 48‐well plate was displayed. G) Numbers of calcium nodules from (F). H,I) VHL cKO mice were intraperitoneally injected with 3 mg/kg α‐NETA every other day starting at 18 days of age for a duration of 4 weeks, after which the femurs were analyzed using micro‐CT. H) Representative radiographs of femurs of 7‐week‐old WT, VHL cKO, and α‐NETA treated VHL cKO mice. I) BV/TV (%) of the femurs in (H). *n* = 3 mice per group. Data are represented as mean ± s.d. Statistical significance was assessed by two‐tailed unpaired Student's *t*‐test (A,C–E,G,I).

To identify the role of chemerin in regulating osteogenesis, we cultured bone marrow MSCs in the presence of chemerin under osteoblast differentiation conditions. Addition of chemerin to MSCs cultured under osteoblast‐induction conditions resulted in elevated expression of *Osx*, *Runx2*, *Alpl*, and *Ocn* in a dose‐dependent manner (**Figure** **8**A). ARS staining indicated that chemerin treatment promoted bone mineralization of bone marrow‐derived MSCs under osteoblast differentiation conditions (Figure 8B,C). It is well documented that the Wnt/β‐catenin signaling pathway plays a pivotal role for bone formation.^[^ ^29^ ^]^ Upon chemerin treatment, we detected obviously elevated expression of Wnt signaling coreceptor *Lrp5*, as well as Wnt signaling‐targeted genes such as *Axin2*, *Myc*, and *Ccnd1* in bone marrow MSCs under osteoblast differentiation conditions (Figure 8D). Accordingly, inhibiting chemerin with a CMKLR1 antagonist α‐NETA blocked the osteogenetic differentiation potential of MSCs from VHL cKO mice, indicated by expression of osteoblast‐specific genes, as well as ARS staining (Figure 8E–G). To examine the role of chemerin/CMKLR1 axis of bone formation in vivo, VHL cKO mice were intraperitoneally injected with α‐NETA every other day for 4 weeks after weaning. Micro‐CT analysis revealed that α‐NETA administration remarkably mitigated the bone mass increase of VHL cKO mice (Figure 8H,I). However, CMKLR1 inhibition failed to reverse the expansion of the LSK progenitor population (Figure S8A, Supporting Information). Similarly, α‐NETA treatment did not alleviate neutrophil infiltration in the spleen (Figure S8B, Supporting Information).

Collectively, our data demonstrated a positive role of adipocyte‐derived chemerin in osteoblast differentiation and bone formation both in vitro and in vivo. This osteogenic effect of adipocyte‐derived chemerin contrasts with previously reported inhibitory roles of chemerin in osteogenesis, suggesting a context‐dependent action of this adipokine.^[^ ^30^, ^31^ ^]^

## 3 Discussion

In this study, we demonstrate VHL‐HIF2α axis in mature adipocytes as a master regulator of systemic homeostasis that orchestrates hematopoiesis and bone formation. We found that VHL deficiency in mature adipocytes result in systemic autoinflammation, hematopoietic dysfunction, fat mass decrease and osteosclerosis. VHL‐deficient adipocytes up‐regulate SCF expression, which drives HSCs to exit quiescence, accelerating their proliferation and differentiation, thus exert a detrimental role for hematopoiesis. Direct blockade of SCF signaling rescues the hematopoietic defects in VHL cKO mice. We further identified adipocyte‐derived chemerin as a critical modulator that links hypoxia‐induced adipocyte dysfunction to pathological osteosclerosis. At the molecular level, we showed that HIF2α directly binds to the HRE sites in the *Rarres2* gene locus. In addition, chemerin inhibition significantly blocks osteosclerosis in VHL cKO mice. Our findings delineate a hypoxia‐driven signaling network in adipocytes that orchestrates crosstalk with HSCs and MSCs to maintain systemic homeostasis.

Previous studies have demonstrated that hypoxia signals in adipose tissue play a key role in the development of metabolic dysfunctions.^[^ ^15^, ^32^ ^]^ HIF1α accumulation in adipocytes or adipocyte progenitors contributes to insulin resistance, adipose tissue inflammation, and fibrosis in obesity.^[^ ^33^, ^34^, ^35^, ^36^, ^37^ ^]^ However, HIF2α in adipocytes plays a protective role against atherosclerosis, cardiomyopathy, high‐fat diet (HFD) induced metabolic dysfunction, as well as regulates thermogenic programming upon cold stimuli.^[^ ^38^, ^39^, ^40^, ^41^ ^]^ *Fabp4*‐Cre‐driven VHL deficiency leads to cardiac hypertrophy, which is regulated by HIF2α‐induced adipose tissue inflammation.^[^ ^41^ ^]^ However, while *Vhl^fl/fl^ Fabp4‐*Cre mice exhibited normal spleen size, we observed noticeable splenomegaly, high bone density, and abnormal hematopoiesis in *Vhl^fl/fl^ Adipoq‐*Cre mice. These phenotypic discrepancies might be explained by the variant expression patterns of Adiponectin and Fabp4. Adiponectin is peculiarly expressed in mature adipocytes and a subset of marrow adipogenic lineage precursors (MALPs), while Fabp4 can also be expressed in macrophages, endothelial cells, and smooth muscle cells except for its expression in adipocytes.^[^ ^19^, ^42^, ^43^ ^]^ In this study, we found that *Adipoq*‐Cre‐driven VHL depletion regulates hematopoiesis and osteogenesis through HIF2α.

BMAs play crucial roles in regulating hematopoietic homeostasis by secreting specific factors that influence the maintenance and reconstitution of HSCs as well as the differentiation of downstream hematopoietic progenitors.^[^ ^2^, ^22^ ^]^ However, the precise effects of BMAs on hematopoiesis remain controversial. Most studies suggest that BMAs exert a negative regulatory role on HSC differentiation. For instance, “fatless” mice devoid of adipocytes or PPAR‐γ inhibitor‐treated mice exhibit accelerated hematopoietic recovery after bone marrow ablation.^[^ ^5^ ^]^ Conversely, in diabetic‐obese (db/db) and HFD‐fed mice, the accumulation of BMAs promotes HSC quiescence and suppressed the proliferation of hematopoietic progenitors.^[^ ^10^, ^11^ ^]^ However, other studies demonstrated that BMAs facilitate hematopoiesis under certain conditions. It has been reported that HFD promotes the expansion and differentiation of HSC cells.^[^ ^44^ ^]^ Also, BMAs are essential for regeneration of hematopoietic progenitors after irradiation through secreting SCF.^[^ ^12^ ^]^ It was reported that lipolysis in bone marrow adipocytes is required for emergency hematopoiesis after myocardial infarction.^[^ ^13^ ^]^ In *Vhl^fl/fl^ Adipoq‐*Cre mice, we observed a marked decrease in bone marrow adipocytes. Intriguingly, VHL‐deficient adipocytes upregulated SCF expression, which was associated with proliferation and differentiation of hematopoietic progenitors, thus resulting in abnormal hematopoiesis and autoinflammation. Our study thus identified a negative role of excessive adipocyte‐derived SCF for hematopoietic homeostasis.

Our hematopoietic regeneration studies employing bone marrow transplantation and 5‐FU treatment establish that the hematopoietic alterations in VHL cKO mice are mediated by niche‐dependent mechanisms rather than cell‐autonomous defects in hematopoietic progenitors. This conclusion supports our model wherein adipocyte VHL‐HIF2α signaling modulates hematopoiesis through SCF. Whether these niche‐derived signals exert a long‐term influence on hematopoietic progenitors remains an important question for future investigation.

Adipocytes are generally considered to exert inhibitory effects on bone formation. Many studies have demonstrated that ablation of adipocytes using different genetic tools or lipodystrophy result in high bone mass.^[^ ^6^, ^7^, ^9^, ^45^ ^]^ In contrast, HFD‐induced obesity or adipogenic committing of MSCs result in bone loss.^[^ ^8^, ^44^, ^46^ ^]^ Additionally, in humans, bone marrow adiposity under pathological conditions such as obesity and aging is generally associated with bone loss and osteoporosis.^[^ ^2^ ^]^ Several studies have revealed that depletion or overexpression of either VHL, HIF1α, HIF2α, or PHD proteins in bone cells cause a variety of skeletal phenotypes, indicating a complicated role of oxygen sensing in different cells for bone health.^[^ ^47^ ^]^ HIF1α is generally considered as a positive regulator of bone formation,^[^ ^48^ ^]^ while the role of HIF2α in osteogenesis is still under debate. Constitutive activation of HIF2α in osteoblasts promotes bone formation.^[^ ^49^ ^]^ However, other studies demonstrated that HIF2α is a negative regulator for osteogenesis^[^ ^50^, ^51^, ^52^ ^]^. Here we originally discovered that VHL‐deficiency in mature adipocytes promotes osteogenesis through HIF2α‐driven chemerin secretion, highlight the crosstalk between hypoxia‐induced adipocyte dysfunction and osteogenesis.

Chemerin is an adipokine that is highly expressed in adipocytes and plays a significant role in metabolic and inflammatory pathologies, as well as in processes such as adipogenesis, angiogenesis, and energy metabolism.^[^ ^27^ ^]^ Elevated levels of chemerin have been observed in individuals with osteoporosis, obesity, type 2 diabetes, and nonalcoholic fatty liver disease.^[^ ^27^, ^53^, ^54^ ^]^ Several studies have defined chemerin as a negative regulator of bone formation. Genetic silencing of chemerin or its receptor CMKLR1 has been shown to result in increased bone mass and mineral density. In vitro knockdown of chemerin or CMKLR1 promotes adipogenesis and thus reciprocally restrains osteoblastogenesis of bone marrow MSCs.^[^ ^30^, ^55^ ^]^ Global genetic ablation of chemerin has demonstrated that it blocks osteogenesis through inhibiting Wnt/β‐catenin signaling while promotes osteoclast differentiation and proliferation via activating RANK signaling.^[^ ^31^ ^]^ Conversely, obesity‐, age‐, and ovariectomy‐induced bone loss have been associated with high serum chemerin levels in clinical studies.^[^ ^56^, ^57^, ^58^ ^]^ However, a recent study reported that chemerin promotes osteogenic differentiation.^[^ ^59^ ^]^ Here, we found that VHL‐deficient mature adipocytes dramatically upregulated chemerin expression through HIF2α and thus promoted osteoblastogenesis. Accordingly, chemerin inhibition rectified the bone mass increase of VHL cKO mice. Our data thus revealed a positive regulatory role of adipocyte‐derived chemerin in bone formation, which appears paradoxical compared to its established role as a negative regulator of osteogenesis.

We propose that this apparent contradiction underscores the exquisite context‐ and source‐dependency of chemerin signaling. While previous studies, relying primarily on in vitro models using MSC cells or systemic knockout approaches, have established chemerin as a negative regulator of bone formation, our work reveals a distinct osteopromotive role for adipocyte‐derived chemerin in vivo. A critical distinction of our model is that the MSCs capable of differentiating into osteoblasts are WT for chemerin and its receptor CMKLR1. This indicates that the pro‐osteogenic effect is not due to an intrinsic change in the MSCs, but rather is driven by a paracrine mechanism originating from the VHL‐deficient adipocytes. The most compelling evidence supporting this novel function is our experimental demonstration that neutralizing CMKLR1 effectively reverses the osteosclerotic phenotype in the VHL cKO model, establishing a direct link for adipocyte‐derived chemerin in promoting bone formation.

While the Adipoq‐Cre driver effectively targets adipocyte‐lineage cells, it is recognized that its activity is not confined to mature adipocytes but also includes MALPs, which constitute less than 10% of total bone marrow stromal cells.^[^ ^43^, ^60^ ^]^ Consequently, the phenotypic outcomes in our VHL cKO model may arise from VHL deletion in both mature adipocytes and MALPs. Importantly, lineage‐tracing studies have established that Adipoq‐Cre‐labeled cells are committed to the adipocyte lineage and rarely give rise to osteoblasts in vivo, nor do they exhibit osteogenic potential in vitro.^[^ ^3^, ^61^ ^]^ These evidences effectively exclude confounding contributions from osteoblast progenitors or the broader MSC population to the observed phenotypes.

In conclusion, we demonstrate that the VHL‐HIF2α axis in adipocytes fine‐tunes hematopoiesis and bone formation through modulating SCF and chemerin expression respectively. We uncover a previously unrecognized role of HIF2α‐driven chemerin secretion in adipocytes in promoting MSC osteoblastogenesis. Our study reveals that hypoxia‐sensing machinery in mature adipocytes orchestrates an endocrine network with HSCs and MSCs to preserve systemic homeostasis, identifying a targetable signaling axis for treating adipocyte dysfunction‐related disorders.

## 4 Experimental Section

### Mice

All mice used in this study were on a C57BL/6 genetic background. *Adipoq‐*Cre, *Vhl^fl/fl^*, *Hif1a^fl/fl^*, and *Hif2a^fl/fl^* mice were purchased from Jackson Laboratory. In all experiments, heterozygous Cre mice were used alongside sex‐matched littermate controls. CD45.1+ mice were from Y. Shi (Tsinghua University). All animal studies were conducted in accordance with and approved by the Institutional Animal Care and Use Committee of Laboratory Animal Resources Center at Tsinghua University. The ethical approval number for our animal studies is 16‐LYC3.

### Isolation of Stromal Vascular Fraction (SVF) and Mature Adipocyte Fraction (AF)

Epididymal white adipose tissue (eWAT) from 6‐ to 8‐week‐old mice was minced using scissors and subsequently digested with Collagenase Type III (1 mg mL^−1^, Worthington) and DNase I (20 µg mL^−1^, Roche) in RPMI 1640 medium at 37 °C with continuous agitation for 30 min. The digested cell suspensions were filtered through 70 µm cell strainers and centrifuged at 1800 rpm for 5 min. The floating cell layer was collected as mature adipocytes, while the pellet was collected as SVF.

### Isolation of Lymphocytes

Bone marrow cells were collected by flushing the femurs and tibias with RPMI‐1640 media with 2% heat‐inactivated bovine serum. For isolation of cells from the spleen and livers, tissues were homogenized manually through 70 µm cell strainers. To enrich lymphocytes in the livers, cell suspensions were enriched with 40% Percoll gradient after red blood cells were lysed. Single‐cell suspensions were used for subsequent flow cytometry staining.

### Antibodies and Reagents

The following fluorochrome‐ or biotin‐conjugated monoclonal antibodies (mAbs) were used for flow cytometry. mAbs specific for mouse CD3 (145‐2C11), CD4 (GK1.5), CD8α (53‐6.7), CD5 (53‐7.3), Gr‐1 (RB6‐8C5), erythroid cell marker (TER‐119), CD19 (1D3), B220 (RA3‐6B2), CD11b (M1/70), CD11c (N418), Ly‐6G (1A8), CD150 (TC15‐12F12.2), CD48 (HM48‐1), and streptavedin were purchased from BioLegend. mAbs to mouse CD45 (30‐F11), CD44 (IM7), CD127 (IL‐7R; A7R34), c‐Kit (2B8), CD45.1 (A20), CD45.2 (104), CD62L (MEL‐14), CD34 (RAM34), CD16/32 (93), IFN‐γ (XMG1.2), and Ki‐67 (SolA15) were from eBioscience. mAb to mouse Sca‐1 (D7) was from BD Biosciences. Hoechst 33342 was from Life technologies. Chemerin was from Peprotech. Pyronin Y and α‐NETA were from Sigma. Anti‐mCD117 monoclonal antibody was from Bioxcell.

### Flow Cytometry

Samples were pre‐incubated with Fc Block (eBioscience) for 10 min. For phenotypic analysis by flow cytometry, samples were run on an LSR Fortessa (BD Biosciences) and data were analyzed using FlowJo (Tree Star). For lineage staining, cells were first stained with biotinylated anti‐mouse CD3, CD4, CD8α, CD5, Gr‐1, TER‐119, CD19, B220, CD11b, CD11c, and Ly‐6G antibodies, and then stained with Brilliant Violet 421 anti‐streptavidin antibody. For sorting of LSKs from bone marrow cells, lineage negative (Lin^–^) cells were firstly enriched by mouse Lineage Cell Depletion Kit (Miltenyi Biotec) following the manufacturer's instructions. Cells were sorted on an Aria II (BD Biosciences).

### Intracellular Staining

For measurement of intracellular cytokine expression, cells were isolated ex vivo and stimulated with 50 ng mL^−1^ PMA and 500 ng mL^−1^ ionomycin, in the presence of GolgiStop (BD Biosciences) for 3 h. Dead cells were excluded by Fixable Viability Dye eFluor 450 (eBioscience). Cells were stained with antibodies to surface antigens, and then fixed and permeabilized with the Cytofix/Cytoperm kit (BD Biosciences), followed by staining with anti‐IFN‐γ (eBioscience). For analysis of transcription factor expression, cells were stained with antibodies to surface antigens, then fixed and permeabilized with the Foxp3/Transcription Factor Staining Buffer Set (eBioscience) according to the manufacturer's instructions, and stained with anti‐Ki‐67.

### Hoechst 33342 and Pyronin Y Staining

For measurement of cell cycle, Lin^−^ enriched bone marrow cells are resuspended in pre‐warmed (37 °C) IMDM at a concentration of 1× 10^6^ per mL. Hoechst dye was added to a final concentration of 10 µg mL^−1^ and incubated at 37 °C for 45 min. Then, Pyronin Y was added directly to cells at a final concentration of 1 µg mL^−1^ and incubated at 37 °C for another 15 min. Cells were washed for once and stained with antibodies to surface antigens.

### BrdU Incorporation

5‐week‐old mice were daily injected with 1 mg BrdU intraperitoneally for five consecutive days. Organs were collected 24 h after the last injection. Cells were surface stained for flow cytometry as described above and then stained for BrdU incorporation using the BD APC BrdU Flow Kit (eBioscience) following the manufacturer's instructions.

### Competitive Reconstitution Assay

Adult CD45.1+ recipient mice were given two doses of 550 rads irradiation (total 1100 rads). Bone marrow cells (1 × 10^6^ each) obtained from WT or *Vhl^fl/fl^ Adipoq*‐Cre (CD45.2+) mice were mixed with equal numbers of bone marrow cells (1 × 10^6^) from CD45.1+ WT mice and then injected into irradiated WT CD45.1+ recipient mice intravenously. After 8 weeks, the spleen and bone marrow were collected for FACS analysis.

### Bone Marrow Regeneration

4‐week‐old CD45.2+ WT or *Vhl^fl/fl^ Adipoq*‐Cre recipient mice were given two doses of 450 rads irradiation (total 900 rads). Bone marrow cells (2 × 10^6^) obtained from WT CD45.1+ mice were injected into irradiated recipient mice intravenously. After 4 weeks, the bone marrow cells were collected for FACS analysis.

### Histology

Bones and other tissues (eWAT and spleen) were fixed in 4% paraformaldehyde overnight. Bones were decalcified in phosphate‐buffered saline (PBS) with 10% EDTA and 30% sucrose for 5 days. Decalcified mouse bones or formalin‐fixed tissues were paraffin‐embedded and sectioned at 5 µm for hematoxylin and eosin staining according to the manufacturers’ instructions. For TRAP staining, TRAP/ALP staining kit (WAKO) was used following manufacturer's instructions. Images were captured on Zeiss Axio Scan. Z1.

### Microcomputed Tomography (micro‐CT)

Femurs of adult mice were fixed with 4% PFA overnight and then stored in 70% Ethanol. Quantum GX micro‐CT system was used for scanning with scanning parameters 70 kV, 114 µA. Scans were reconstructed and analyzed using Analyze 12.0 software (AnalyzeDirect, Inc.) according to standardized protocol.

### Immunofluorescence Staining

Epididymal white adipose tissue (eWAT) samples were fixed overnight in formalin and embedded in paraffin. Sections (4 µm) were deparaffinized in xylene and rehydrated through a graded ethanol series. Antigen retrieval was performed using sodium citrate buffer (10 × 10^−3^ m sodium citrate, 0.05% Tween 20, pH 6.0) via microwave heating. Sections were blocked with 10% normal donkey serum and incubated overnight at 4 °C with primary antibodies against HIF2α (Abcam, ab109616) and CD36 (Abcam, ab252922) diluted in antibody dilution buffer containing 0.3% Triton X‐100 and 1% BSA. After washing, sections were incubated with species‐matched HRP‐conjugated secondary antibodies for 50 min at room temperature, followed by tyramide signal amplification (TSA) using fluorescent dyes. Nuclei were counterstained with DAPI. Images were acquired using an Leica STELLARIS 8 Falcon confocal microscope and analyzed with ImageJ software.

### 5‐Fluorouracil‐Induced Emergency Hematopoiesis Model

To assess hematopoietic stress response, mice received a single intraperitoneal injection of 5‐fluorouracil (5‐FU; Sigma‐Aldrich, F6627) at a dose of 150 mg kg^−1^ body weight. Hematopoietic analysis was performed 10 days post‐injection to evaluate regeneration during the recovery phase.

### Isolation of Primary Bone Marrow Adipocytes

Femurs and tibias were cut into 1 mm length sections with scissors and digested with Collagenase Type 3 (2 mg mL^−1^, Worthington) and DNase 1 (40 ng mL^−1^, Roche) in RPMI 1640 at 37 °C with agitation for 20 min. Centrifuge at 200 × *g* for 10 min to separate adipocytes (floating layer) from stromal cells and bone debris. Carefully transfer the floating adipocyte layer to a new tube using a wide‐bore pipette. Wash twice with PBS by gentle centrifugation.

### Isolation and Culture of Bone Marrow‐Derived MSCs

Femurs and tibias were cut into 1 mm length sections with scissors and digested with Collagenase Type 3 (2 mg mL^−1^, Worthington) and DNase 1 (40 ng mL, Roche) in RPMI 1640 at 37 °C with agitation for 40 min. For adipocyte differentiation, digested cells were collected and stained with biotin‐conjugated anti‐CD45 and anti‐CD31 antibodies. CD45^−^CD31^−^ bone marrow cells were enriched through negative magnetic separation (Miltenyi Biotec) and seeded into 6‐well plates; for osteoblast differentiation, total digested cells and bone debris were directly seeded into 6 cm culture dishes. Cells were cultured in complete MSC culture medium, consisting of MEM‐alpha (Gibco) supplemented with 15% heat‐inactivated FBS, 55 × 10^−6^ m β‐mercaptoethanol (Gibco), and 1% penicillin/streptomycin. Non‐adherent cells were removed after 4 h, and the medium was replaced every 2 days until confluent. Adherent cells were passaged and maintained for up to two passages as BM MSCs. To remove bone debris, the digested cell suspension was filtered through 70 µm cell strainers. For differentiation assays, MSCs were seeded into 24‐well or 48‐well plates and cultured in DMEM (Gibco) supplemented with 10% heat‐inactivated FBS and 1% penicillin/streptomycin, along with either adipocyte‐induction or osteoblast‐induction chemicals.

### Isolation and Culture of Adipose‐Derived MSCs

eWATs were minced and digested with Collagenase Type III and DNase I at 37 °C with continuous agitation for 20 min. The digested mixture was centrifuged at 400 × g for 5 min at 4 °C, and the pellet was resuspended and seeded into 6 cm culture dishes and cultured with complete MSC culture medium. Non‐adherent cells were removed after 4 h, and the medium was replaced every 2 days until confluent. Cells were passaged and maintained for up to two passages as adipose‐derived MSCs.

### MSC Adipogenic Differentiation and Oil Red O Staining

To induce adipogenic differentiation, MSCs or OP9 cells were cultured in adipogenic‐inducing medium containing 0.5 × 10^−3^ m isobutylmethylxanthine (IBMX, Sigma‐Aldrich), 5 µg mL^−1^ insulin (Sigma‐Aldrich), 1 × 10^−6^ m dexamethasone, and 1 × 10^−6^ m pioglitazone (Sigma‐Aldrich). On day 3, the medium was replaced with maintenance medium containing 5 µg mL^−1^ insulin, and then refreshed on day 5. On day 7, the medium was replaced with maintenance medium without insulin. For Oil Red O staining, cells were washed with PBS and fixed with 4% paraformaldehyde for 10 min, followed by washing with ddH2O. Oil Red O (0.5% in isopropanol) was diluted with water (3:2 ratio), filtered through a 0.45 µm filter, and incubated with the fixed cells for 30 min at room temperature with gentle shaking. Cells were then washed for 4 times with ddH2O, and the stained lipid droplets were visualized and photographed using light microscopy.

### MSC Osteoblastic Differentiation and ARS Staining

To induce osteogenic differentiation, bone marrow MSCs or OP9 cells were cultured in osteoblast‐inducing medium supplemented with 100 × 10^−6^ m ascorbic acid, 2 × 10^−3^ m β‐glycerophosphate, and 10 × 10^−9^ m dexamethasone (TAKARA). The medium was replaced every 2–3 days. After 14–21 days, cells were fixed for Alizarin Red S staining. Briefly, cells differentiated in 48 well plate were fixed with 4% paraformaldehyde for 10 min and washed with ddH2O. Fixed cells were stained with 2% Alizarin red S (Sigma–Aldrich) solution for 30 min at room temperature and washed with ddH2O for three times.

### Retroviral Transduction

For retroviral transduction, shRNA‐containing oligonucleotides (shScramble: TGCTGTTGACAGTGAGCGACCATAGATGTTACCCTTTATTTAGTGAAGCCACAGATGTAAATAAAGGGTAACATCTATGGCTGCCTACTGCCTCGGA; and shVhl: TGCTGTTGACAGTGAGCGCGCTGTTTGTGCCATCCCTCAATAGTGAAGCCACAGATGTATTGAGGGATGGCACAAACAGCTTGCCTACTGCCTCGGA) were cloned into the LMP vector according to the manufacturer's protocol (Open Biosystems). Plat‐E packaging cells cultured in 6 well plate were transfected with 3 µg of retroviral vector along with 9 µl of TransIT‐LT1 transfection reagent (Mirus). The culture supernatant containing retrovirus was collected 48 h after transfection and used to transduce OP9 cells. 72 h after transduction, Ametrine^+^ cells were enriched through FACS sorting to get a stable shRNA knockdown cell line.

### In Vitro LSK Culture

For the LSK culture assay, shScramble or shVhl OP9 cells were treated with adipogenic medium for 7 days to obtain control or VHL‐deficient adipocytes. On day 7, the medium was replaced with StemSpan SFEM (STEMCELL) medium and the supernatant was collected on day 9. LSKs isolated from the bone marrow of WT mice were cultured at a density of 5000–10000 cells per well with OP9‐Adi supernatant supplemented with 5 ng mL^−1^ SCF (Peprotech) and 5 ng mL^−1^ TPO (Peprotech). At day 3, cells were collected for FACS analysis.

### ELISA

For detection of autoantibodies, chemerin, or SCF, serum was collected from male mice at 3 or 8 weeks of age. Antibodies to double‐stranded DNA or histone were measured as previously described.^[^ ^62^, ^63^ ^]^ Chemerin was measured using Mouse Retinoic acid receptor responder protein 2 (RARRES2) ELISA kit according to the manufacturers’ instructions (CUSABIO). SCF levels were determined using a specific murine ELISA kit (Solarbio, SEKM‐0131).

### Real‐Time Quantitative PCR

For real‐time quantitative PCR analysis, total RNA was extracted with Trizol (Thermo Scientific) and reversely transcribed to cDNA with RevertAid First Strand cDNA Synthesis Kit (Thermo Scientific). Quantitative real‐time PCR was performed using iQ SYBR Green Supermix (Bio‐Rad) in triplicates with a CFX96 real‐time system (Bio‐Rad). Samples were normalized to the expression of gene encoding β‐actin. The primer sequences used in this study were listed in Table S1 (Supporting Information).

### RNA‐Based Next‐Generation Sequencing (RNA‐seq)

Total RNA of mature adipocytes from eWAT of 8‐week‐old male mice was prepared by RNeasy mini kit (Qiagen) with RNase free DNase set (Qiagen). Sample preparation, library construction, and sequencing on BGISEQ‐500 were performed at Beijing Genomics Institute at 50‐bp read length. Analysis was undertaken by Beijing Genomics Institute and our laboratory.

### Lipidomics

Fresh isolate mature adipocytes from eWAT or serum of 8‐week‐old male mice were used for lipid extraction by methanol and CH2Cl2. The adipocyte counts were normalized by BCA Protein Assay Kit (Pierce). After drying down in an argon evaporator, extracted lipids were dissolved and loaded for LC‐MS assay at the Facility Center of Metabolomics and Lipidomics, National Center for Protein Sciences, Tsinghua University.

### Chromatin Immunoprecipitation (ChIP) Assay

ChIP was performed as described in previous reports with some modifications.^[^ ^64^, ^65^ ^]^ Briefly, adipose‐derived MSCs from eWAT of WT or *Vhl^fl/fl^Adipoq*‐Cre mice were induced into adipocytes. Cells were collected and treated with hypotonic buffer before fixation. Lysed cells were sonicated with Bioruptor using 30 cycles of 30 s on/off. After spinning at max speed at 4 °C for 15 min, the supernatant was transferred to a new tube, and 10% was kept as input for later purification. Each chromatin sample was incubated with 2 µg HIF‐2 alpha (Novus Biologicals, NB100‐122), or 2 µg H3K4me3 (Abcam, ab8580), or 2 µg rabbit isotype control IgG (Abcam, ab171870) antibodies at 4 °C overnight. The samples were incubated with 40 µl protein A/G dynabeads (Life Technologies) for another 4 h. The beads were washed with wash buffer and then resuspended with TE buffer supplemented with 50 µg mL^−1^ proteinase K (Roche) and incubated at 55 °C with shaking for 4 h to digest proteins. The supernatant was collected and DNA was purified by phenol‐chloroform extraction and ethanol precipitation. Enrichment of histone modifications were analyzed by RT‐PCR using validated primers listed in Table S2 (Supporting Information). The ratio of precipitated DNA to input DNA was calculated for comparison of histone modification levels between different samples.

### Statistical Analysis

All statistical analyses were conducted using GraphPad Prism 9.0. Data collection and analysis were performed blind to the experimental conditions if possible. All values are given as mean ± s.d. and all *n* values represent biological replicates or technical replicates. Results are presented for experiments using at least three biological replicates and *p* values < 0.05 were considered statistically significant.

## Conflict of Interest

The authors declare no conflict of interest.

## Author Contributions

Q.L., J.L., and A.T. contributed equally to this work. Y.‐C.L. and Q.L. conceived the study. Q.L. and J.L. designed experiments. Q.L., J.L., and A.T. conducted experiments and analyzed the data. C.L. performed histological analysis and lipidomics. C.Z. and Q.L. performed bioinformatics analysis. C.Z. helped mice analysis. Q.L. and J.L. visualized the data and wrote the original draft of the manuscript. Q.L. and Y.‐C.L. reviewed and edited the manuscript. Y.‐C.L. supervised the study.

## Supporting information



Supporting Information

## Data Availability

The data that support the findings of this study are available from the corresponding author upon reasonable request.
