# Supplementary material for: Bifurcate Regulation of Hematopoietic Homeostasis and Bone Osteogenesis by VHL‐HIF2α‐Controlled Adipocyte Function
Source: Adv Sci (Weinh). 2025 Nov 8;13(5):e09255. doi: 10.1002/advs.202509255 (PMC12850111; doi:10.1002/advs.202509255)
Supplement: Supplementary file 1 — Supporting Information [file ADVS-13-e09255-s001.pdf]

Supporting Information

Bifurcate regulation of hematopoietic homeostasis and bone osteogenesis by VHL-HIF2α-controlled adipocyte function

This file includes:

Figure S1 to S8

Tables S1 to S2

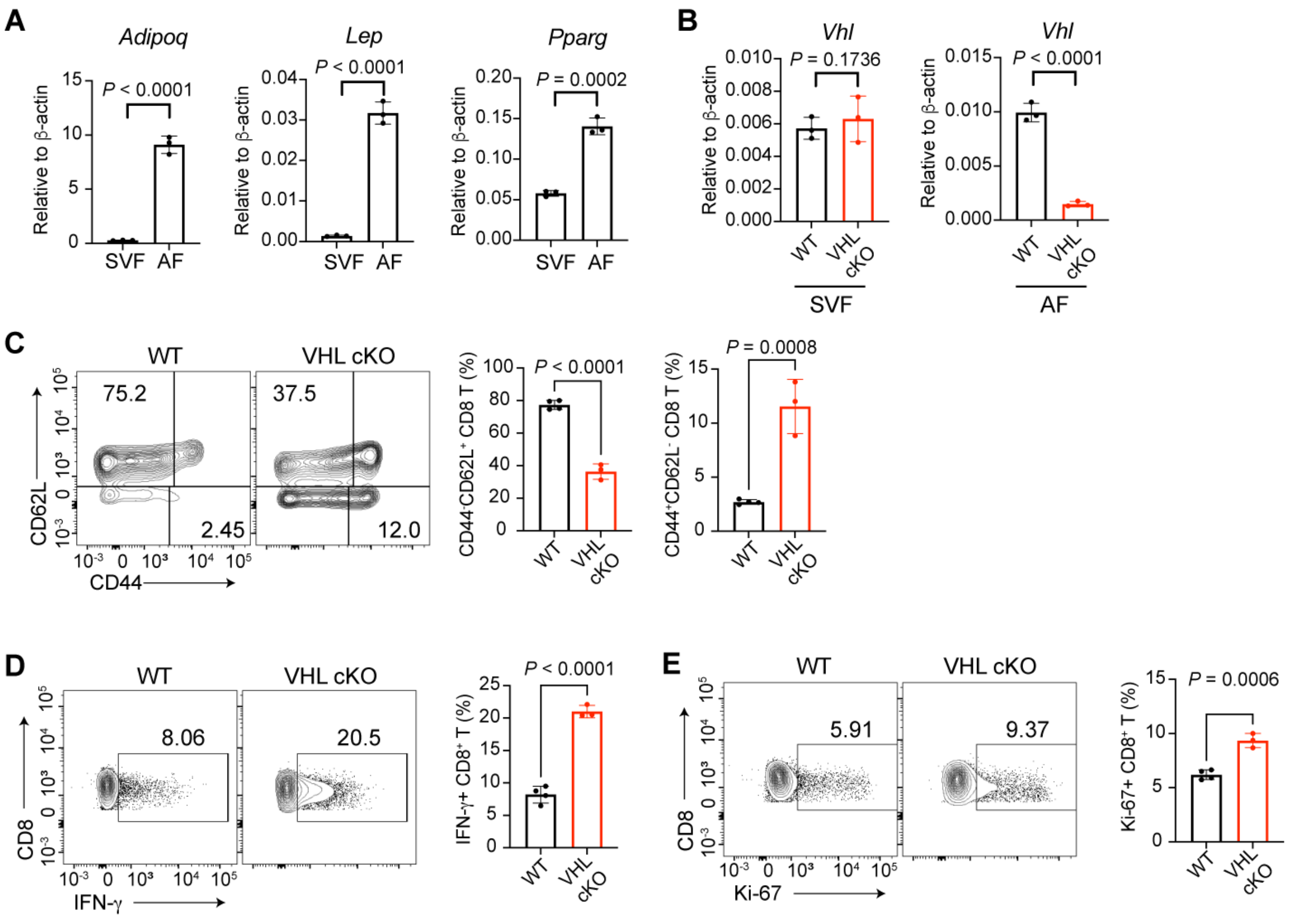

Figure S1. Von Hippel-Lindau (VHL) deficiency in mature adipocytes results in systemic autoinflammation. A) mRNA expression of *Adipoq*, *Lep*, and *Pparg* in the stromal vascular fraction (SVF) and adipose fraction (AF) of eWAT from wild-type mice. B) mRNA expression of *Vhl* in the SVF and AF of wild-type (WT) and VHL cKO mice. C) Flow cytometric analysis (left) and frequencies (right) of naive ($CD44^{-}CD62L^{hi}$) $CD8^{+}$ T cells and effector-like ($CD44^{hi}CD62L^{-}$) $CD8^{+}$ T cells from peripheral blood of WT and VHL cKO mice. n = 3-4 mice per group. D and E) Flow cytometric analysis (left) and frequencies (right) of IFN-γ^{+} (D) and Ki-67^{+} (E) $CD8^{+}$ T cells from peripheral blood of WT and VHL cKO mice. n = 3-4 mice per group. Data are represented as mean ± s.d. Statistical significance was assessed by two-tailed unpaired Student's *t*-test (A-E).

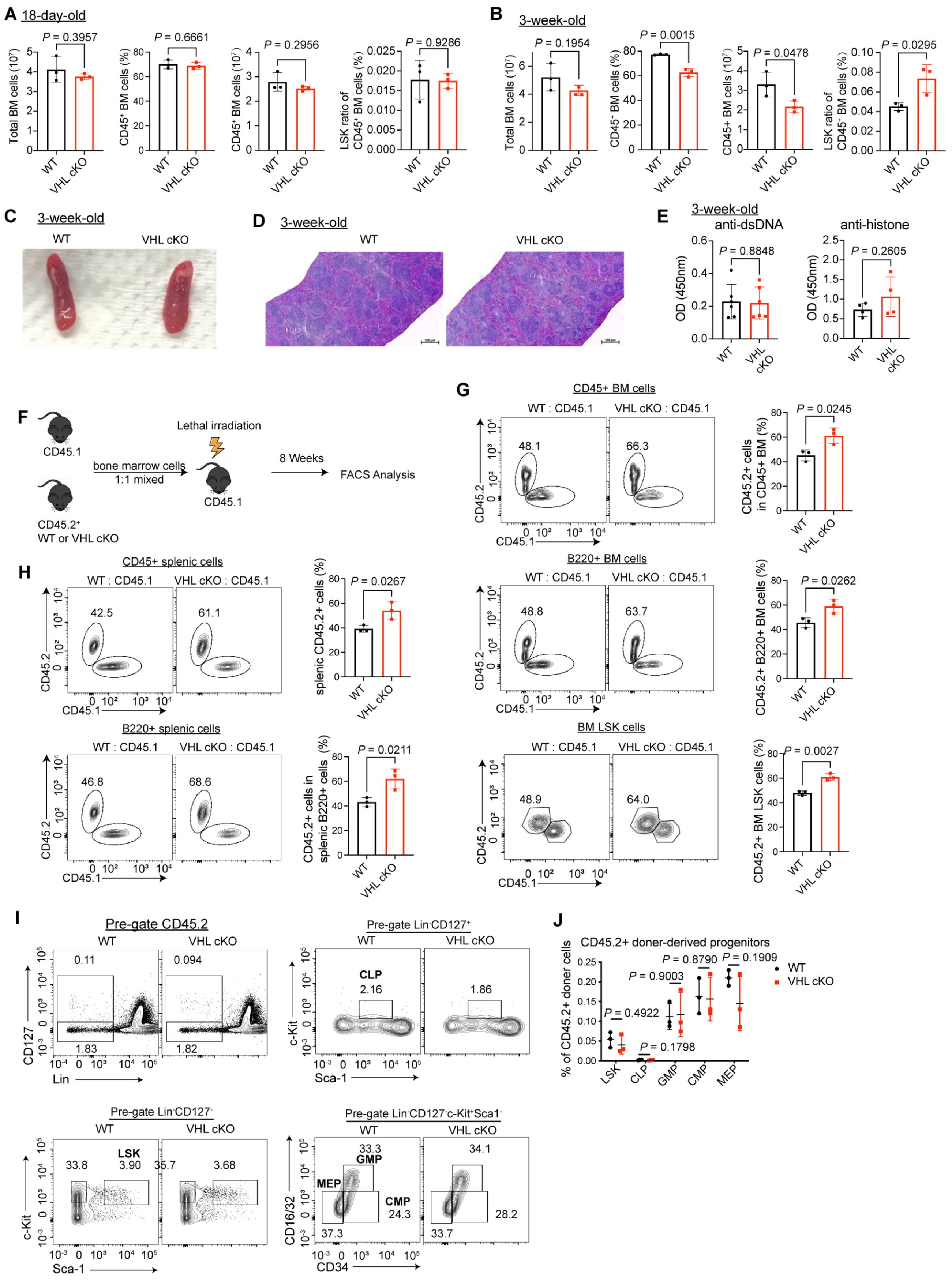
A 18-day-old
P = 0.3957
P = 0.6661
P = 0.2956
P = 0.9286
Total BM cells (10⁷)
CD45⁺ BM cells (%)
CD45⁺ BM cells (10⁷)
LSK ratio of CD45⁺ BM cells (%)
WT
VHL cKO
B 3-week-old
P = 0.1954
P = 0.0015
P = 0.0478
P = 0.0295
CD45+ BM cells (10⁷)
C 3-week-old
D 3-week-old
E 3-week-old
anti-dsDNA
anti-histone
P = 0.8848
P = 0.2605
OD (450nm)
F
CD45.1
CD45.2⁺
WT or VHL cKO
bone marrow cells
1:1 mixed
Lethal irradiation
8 Weeks
FACS Analysis
G
CD45+ BM cells
WT : CD45.1
VHL cKO : CD45.1
48.1
66.3
P = 0.0245
CD45.2+ cells in CD45+ BM (%)
H
CD45+ splenic cells
42.5
61.1
P = 0.0267
splenic CD45.2+ cells (%)
B220+ BM cells
48.8
63.7
P = 0.0262
CD45.2+ B220+ BM cells (%)
B220+ splenic cells
46.8
68.6
P = 0.0211
CD45.2+ cells in splenic B220+ cells (%)
BM LSK cells
48.9
64.0
P = 0.0027
CD45.2+ BM LSK cells (%)
CD45.2
CD45.1
I
Pre-gate CD45.2
0.11
0.094
1.83
1.82
CD127
Lin
Pre-gate Lin⁻CD127⁺
CLP
2.16
1.86
c-Kit
Sca-1
Pre-gate Lin⁻CD127⁻
LSK
33.8
3.90
35.7
3.68
Pre-gate Lin⁻CD127⁻c-Kit⁺Sca1⁻
33.3
GMP
MEP
CMP
24.3
37.3
34.1
28.2
33.7
CD16/32
CD34
J
CD45.2+ doner-derived progenitors
% of CD45.2+ doner cells
P = 0.4922
P = 0.1798
P = 0.9003
P = 0.8790
P = 0.1909
LSK
CLP
GMP
CMP
MEP

Figure S2. Von Hippel-Lindau (VHL) ablation in adipocytes attenuates bone marrow hematopoiesis. A and B) Absolute numbers of total bone marrow cells, ratios of $CD45^+$ bone marrow cells, absolute numbers of $CD45^+$ bone marrow cells, ratios of bone marrow LSK cells from 18-day-old (A) or 3-week-old (B) WT and VHL cKO mice. *n* = 3 mice per group. C) Representative image of spleens from 3-week-old WT and VHL cKO mice. D) H&E staining of spleen sections of 3-week-old WT and VHL cKO mice. Scale bars: 500 µm. E) ELISA of antibodies to double-stranded DNA (anti-dsDNA) and histone (anti-histone) in the serum of 3-week-old male mice, presented as optical density at 450 nm (OD_{450}). *n* = 4-6 mice per group. F) Schematic diagram of mix bone marrow transfer. G) Flow cytometric analysis and quantification of $CD45.2^+$ cells among total $CD45^+$ cells, $B220^+$ cells, and LSK cells in the bone marrow of recipient mice. H) Flow cytometric analysis and quantification of $CD45.2^+$ cells among total $CD45^+$ cells and $B220^+$ cells in the spleen of recipient mice. *n* = 3 mice per group. I) Flow cytometric analysis of $CD45.2^+$ LSKs, CLPs, CMPs, GMPs, and MEPs among total $CD45.2^+$ cells in the bone marrow of recipient mice. *n* = 3 mice per group. J) Quantification of $CD45.2^+$ doner-derived progenitors. Data are represented as mean ± s.d. Statistical significance was assessed by two-tailed unpaired Student's *t*-test (A, B, E, G and H) or multiple unpaired *t*-tests (J).

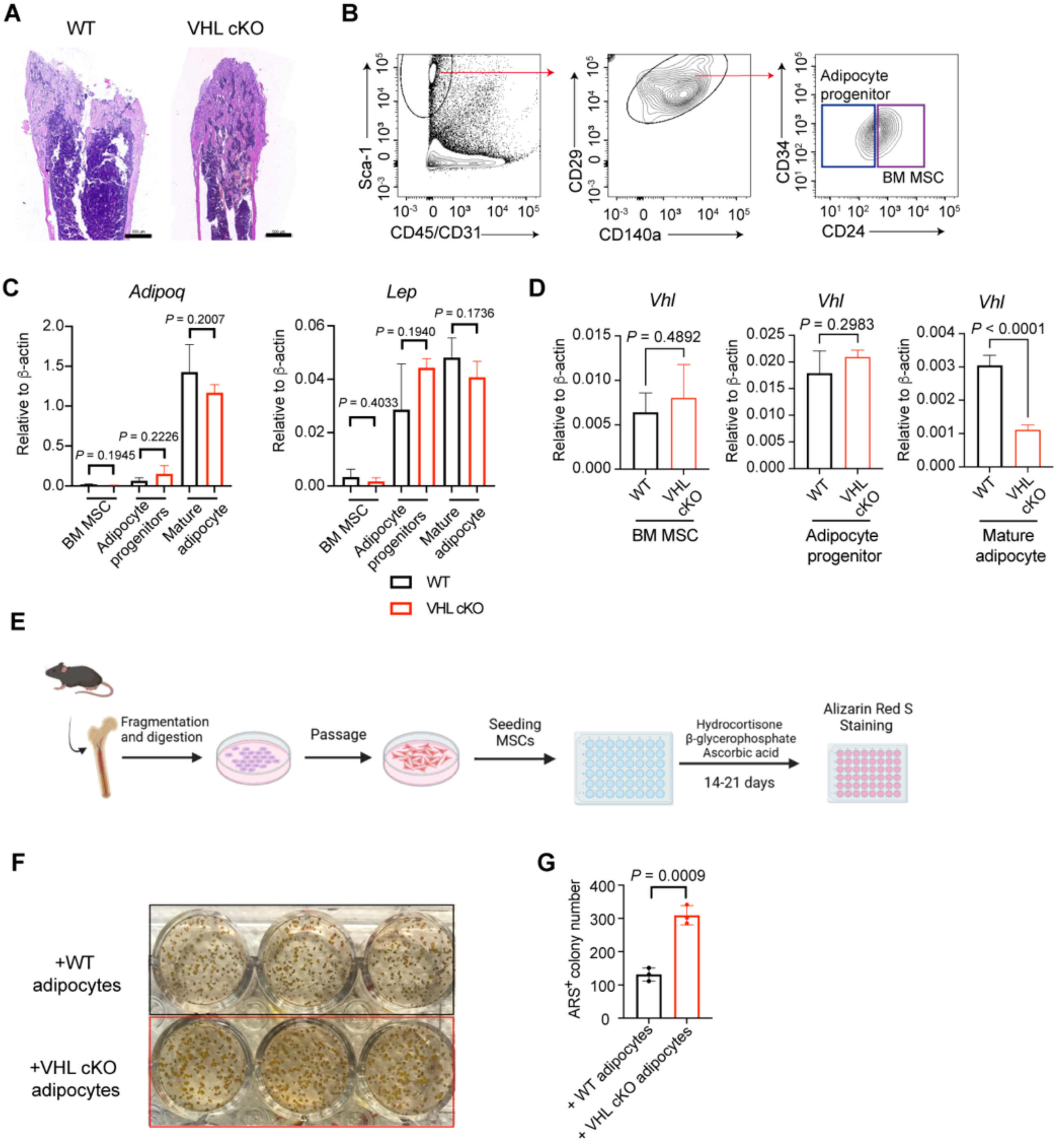

Figure S3. Von Hippel-Lindau (VHL) deficient adipocytes promote osteogenesis. A) Representative H&E staining of femur sections of 3-week-old WT and VHL cKO mice. Scale bars: 500 μm. B) Gating Strategy of MSCs (Purple) and adipocyte progenitors (Blue) from enriched $CD45^{-}$ bone marrow cells. C) mRNA expression of *Adipoq* and *Lep* in sorted bone marrow MSCs, adipocyte progenitors, and eWAT-derived mature adipocytes from of WT or VHL cKO mice. D) mRNA expression of *Vhl* in sorted bone marrow MSCs, adipocyte progenitors, and eWAT-derived mature adipocytes from eWAT of WT or VHL cKO mice. E) Schematic diagram illustrating the osteoblastic differentiation of bone marrow-derived MSCs. F) OP9 cells were co-cultured with fresh isolated mature adipocytes from eWAT of 4-week-old WT or VHL-deficient mice for 3 days, followed by osteoblast-induction

treatment for 14-21 days. The representative captured image indicated ARS staining of OP9-derived osteoblasts. G) Colony numbers of ARS⁺ osteoblasts in (F). Data are represented as mean ± s.d. Statistical significance was assessed by two-tailed unpaired Student's *t*-test (D and G) or multiple unpaired *t*-tests (C).

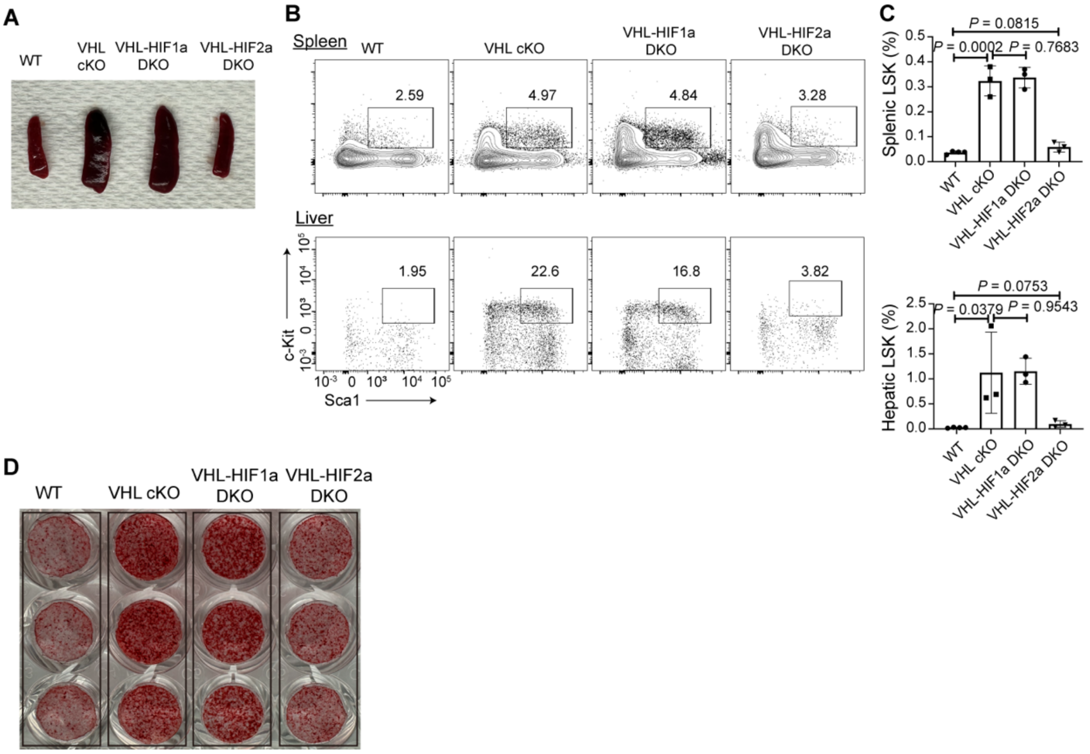

Figure S4. VHL-HIF2α (VHL is Von Hippel-Lindau) axis in adipocytes regulates systemic homeostasis. A) Representative image of spleens from 8-week-old WT, VHL cKO, VHL-HIF1α DKO, and VHL-HIF2α DKO mice. B) Flow cytometric analysis of LSKs in pre-gated Lin⁻CD127⁻ cells from the spleen and liver of WT, VHL cKO, VHL-HIF1α DKO, and VHL-HIF2α DKO mice. C) Frequencies and absolute numbers of LSK cells from the spleen and livers of WT, VHL cKO, VHL-HIF1α DKO and VHL-HIF2α DKO mice. *n* = 3-4 mice per group. D) ARS staining of bone marrow MSC-derived cells from WT, VHL cKO, VHL-HIF1α DKO, and VHL-HIF2α DKO mice under osteoblast-induction conditions. The representative captured image of 48-well plate was displayed. Data are represented as mean ± s.d. Statistical significance was assessed by two-tailed unpaired Student's *t*-test (C).

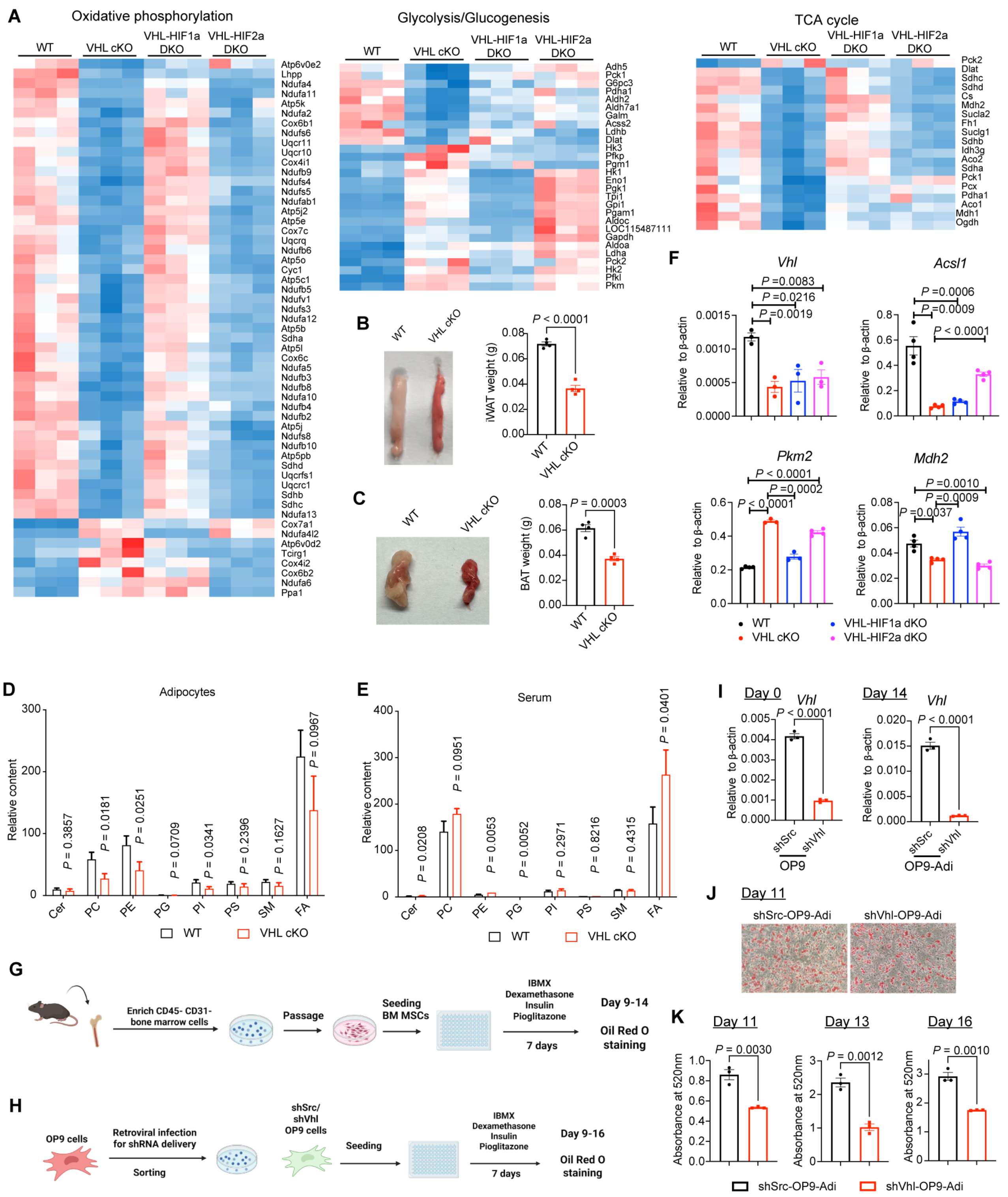

Figure S5. Von Hippel-Lindau (VHL) deletion in adipocytes alters cell metabolism and regulates adipogenesis. A) Heatmaps of differentially expressed genes related to oxidative phosphorylation, glycolysis/glucogenesis, and TCA cycle in mature adipocytes of WT, VHL cKO, VHL-HIF1α DKO, and VHL-HIF2α DKO mice. B) Representative image and weights of iWAT from 6-week-old WT and VHL cKO mice. C) Representative image and weights of BAT from 6-week-old WT and VHL cKO mice. D and E) Relative content of lipid components in mature adipocytes from the eWAT (D) or serum (E) of 8-week-old WT or VHL cKO mice. Cer (Ceramide), PC (Phosphatidylcholine), PE

(Phosphatidylethanolamine), PG (Phosphatidylglycerol), PI (Phosphatidylinositol), PS (Phosphatidylserine), SM (Sphingomyelin) and FA (Fatty acid). F) mRNA expression of *Vhl*, *Acsl1*, *Pkm2* and *Mdh2* in mature adipocytes isolated from the bone marrow of WT, VHL cKO, VHL-HIF1α DKO, and VHL-HIF2α DKO mice. G) Schematic diagram illustrating the adipogenic differentiation of bone marrow-derived mesenchymal stem cells (MSCs). H) Schematic diagram illustrating the adipogenic differentiation of shSrc or shVhl-targeting OP9 cells. I) mRNA expression of *Vhl* in OP9 cells under adipocyte-induction conditions. Cells were defined as OP9 cells on day 0, or as OP9-derived adipocytes (OP9-Adi) on day 14. J) Representative Oil Red O staining of OP9-Adi under adipocyte-induction conditions on day 11. K) Absorbance at 520 nm was measured to quantify Oil Red O staining in OP9-Adi cells with shSrc or shVhl at days 11, 13, and 16. Data are represented as mean ± s.d. Statistical significance was assessed by two-tailed unpaired Student's *t*-test (B,C,F,I and K) or multiple unpaired t-tests (D,E).

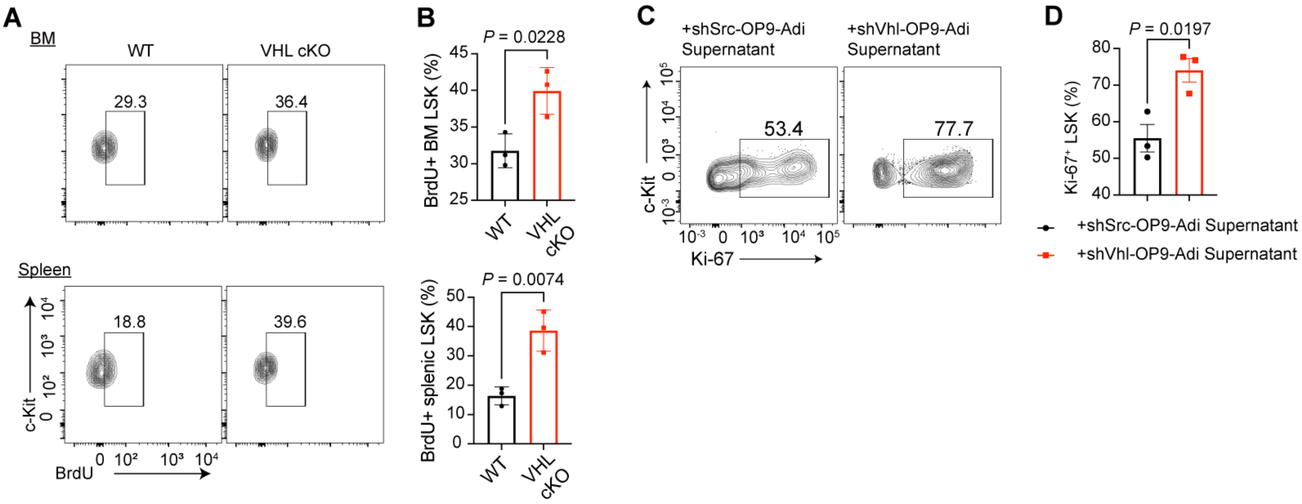

Figure S6. Von Hippel-Lindau (VHL) deletion in adipocytes promote LSK proliferation. A) Flow cytometric analysis of BrdU incorporation in LSK cells from the bone marrow and spleen of WT and VHL cKO mice. B) Frequencies of $BrdU^+$ LSK cells in the bone marrow and spleen of WT and VHL cKO mice. n = 3 mice per group. C and D) FACS sorted LSKs were cultured with supernatants from shScramble (shSrc) or shVhl-targeting OP9-derived adipocytes (OP9-Adi) and analyzed on day 3. C) Flow cytometric analysis of Ki-67 staining in *in vitro* cultured LSK cells. D) Frequencies of Ki-67^+ LSK cells. Data are represented as mean ± s.d. Statistical significance was assessed by two-tailed unpaired Student's *t*-test (B and D).

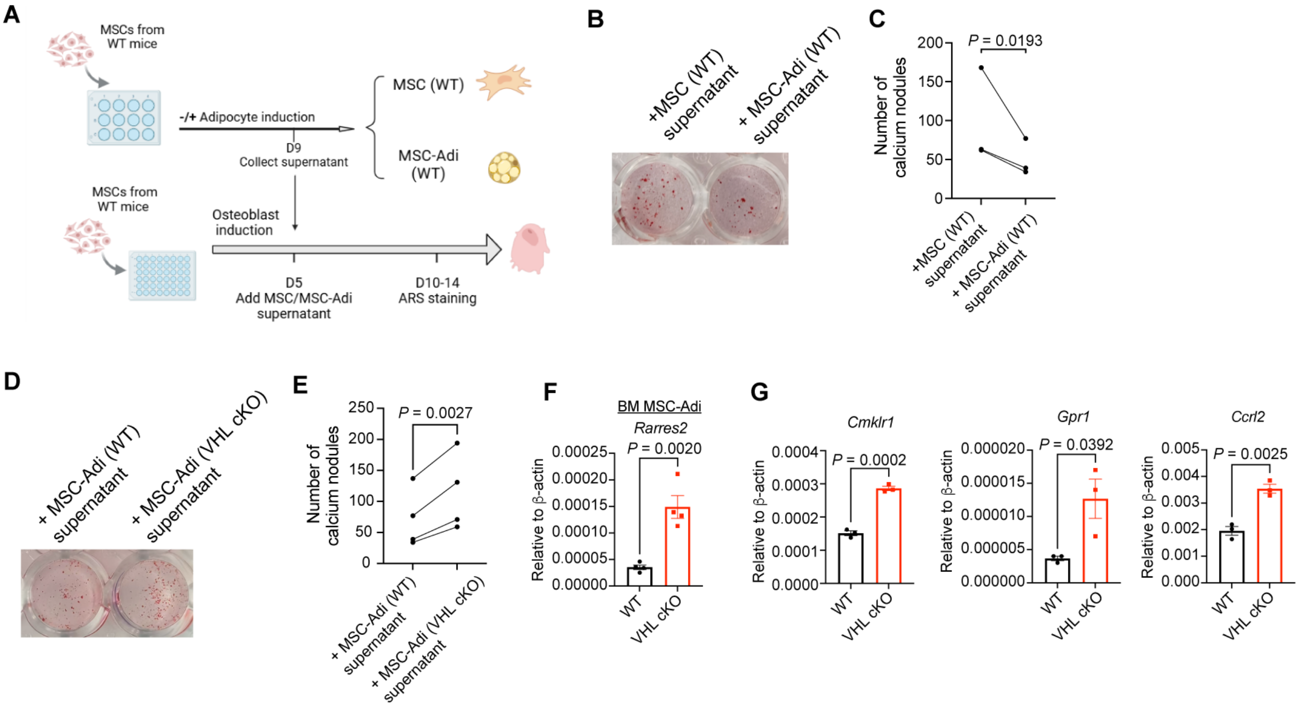

Figure S7. Von Hippel-Lindau (VHL) deficient adipocytes promote MSC osteogenesis through secreted factors. A) Schematic diagram of MSCs cultured with bone marrow MSC supernatants or MSC-derived adipocyte (MSC-Adi) supernatants. B) ARS staining of MSCs cultured in osteoblast-induction medium, supplemented with supernatants from MSCs cultured in common DMEM medium or under adipocyte-induction conditions. The representative captured image of 48-well plate was displayed. C) Numbers of ARS^{+} calcium nodules in (B). D) ARS staining of MSCs cultured in osteoblast-induction medium, supplemented with supernatants from MSC-Adi of WT or VHL cKO mice. The representative captured image of 48-well plate was displayed. E) Numbers of calcium nodules in (D). F) mRNA expression of *Rarres2* in bone marrow MSC-derived adipocytes (BM MSC-Adi) from WT and VHL cKO mice. G) mRNA expression of *Cmklr1*, *Gpr1*, and *Ccrl2* in bone marrow-derived MSCs of WT and VHL cKO mice under osteoblast differentiation conditions on day 8. Data are represented as mean ± s.d. Statistical significance was assessed by two-tailed unpaired Student's *t*-test (C, E-G).

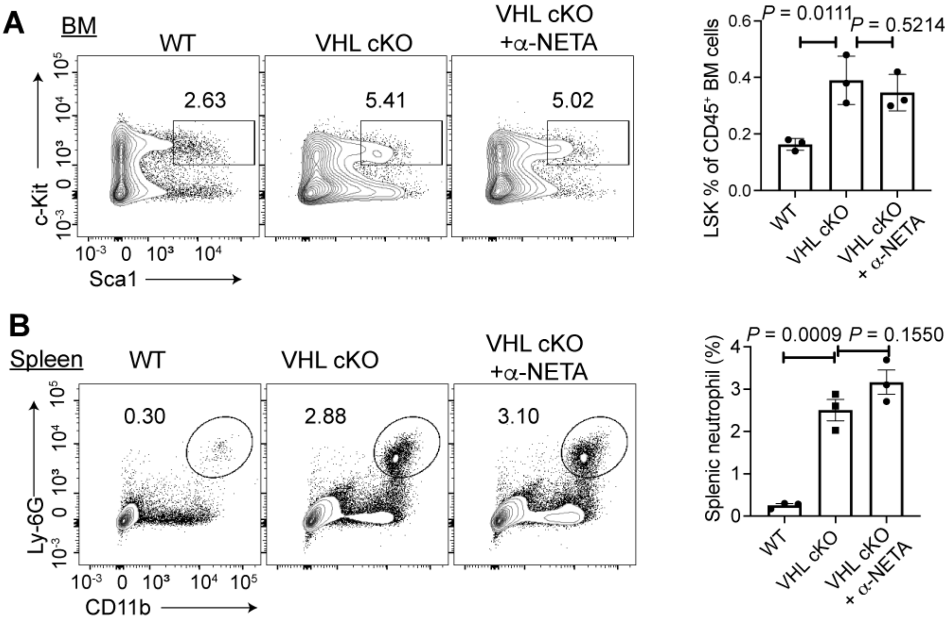

Figure S8. Chemerin promotes bone marrow MSC osteoblastogenesis. VHL cKO mice were intraperitoneally injected with 3 mg/kg α-NETA every other day starting at 18 days of age for a duration of 4 weeks. A) Flow cytometric analysis and frequencies of LSK cells in the bone marrow across indicated genotypes and treatments. $n = 3$ mice per group. B) Flow cytometric analysis and frequencies of neutrophils in the spleen in indicated mice. $n = 3$ mice per group. Data are represented as mean ± s.d. Statistical significance was assessed by two-tailed unpaired Student's *t*-test (A,B).

Table S1. Primers used for quantitative real-time PCR

Gene	Forward primer (5′→3′)	Reverse primer (5′→3′)
Vhl	CAGCTACCGAGGTCATCTTTG	CTGTCCATCGACATTGAGGGA
Adipoq	TGTTCCTCTTAATCCTGCCCA	CCAACCTGCACAAGTTCCCTT
Leptin	GAGACCCCTGTGTCGGTTC	CTGCGTGTGTGAAATGTCATTG
Pparg	GGAAGACCACTCGCATTCCTT	GTAATCAGCAACCATTGGGTCA
Rankl	CTGGGCCAAGATCTCTAACATGA	GGTACGCTTCCCGATGTTTC
Opg	GAGGAGTCTGGTAGTGGTTCC	TGTTCACCCTGGTTGAGTAAGT
Osx	ATGGCGTCCTCTCTGCTTG	TGAAAGGTCAGCGTATGGCTT
Runx2	TTCAACGATCTGAGATTTGTGGG	GGATGAGGAATGCGCCCTA
Alpl	CCAACTCTTTTGTGCCAGAGA	GGCTACATTGGTGTTGAGCTTTT
Ocn	CTGACCTCACAGATCCCAAGC	TGGTCTGATAGCTCGTCACAAG
Col1a1	GCTCCTCTTAGGGGCCACT	CCACGTCTCACCATTGGGG
Wisp-1	CAGCACCACTAGAGGAAACGA	CTGGGCACATATCTTACAGCATT
Scf	GAATCTCCGAAGAGGCCAGAA	GCTGCAACAGGGGGTAACAT
Ckit	GCCACGTCTCAGCCATCTG	GTCGCCAGCTTCAACTATTAACT
Rarres2	CTTCTCCCGTTTGGTTTGATTG	TACAGGTGGCTCTGGAGGAGTTC
Cmklr1	ATGGAGTACGACGCTTACAACG	GGTGGCGATGACAATCACCA
Gpr1	CCCTGTTCTTATATGCCCTAGCA	GCCAGATTGAGGAACCAAAGAGT
Cclr2	GCCCCGGACGATGAATATGAT	CACCAAGATAAACACCGCCAG
Axin2	TGACTCTCCTTCCAGATCCCA	TGCCCACACTAGGCTGACA
Lrp5	AAGGGTGCTGTGTACTGGAC	AGAAGAGAACCTTACGGGACG
Myc	ATGCCCCTCAACGTGAACTTC	GTCGCAGATGAAATAGGGCTG
Ccnd1	GCGTACCCTGACACCAATCTC	CTCCTCTTCGCACTTCTGCTC
β-actin	GCTGTGCTGTCCCTGTATGCCTCT	CCTCTCAGCTGTGGTGGTGAAGC
Acsl1	TGCCAGAGCTGATTGACATTC	GGCATACCAGAAGGTGGTGAG
Pkm2	TTGCAGCTATTCGAGGAACTCCG	CACGATAATGGCCCCACTGC
Mdh2	TTGGGCAACCCCTTTCACTC	GCCTTTCACA TTTGCTCTGGTC

Table S2. Primers for ChIP-qPCR

Gene	Forward primer (5′→3′)	Reverse primer (5′→3′)
P1	TGCTGGTATCCTCAGAGTGC	CTGGGAACCTCAGGGAGAGT
P2	TAACAACAGTCTGGGCAGGA	TCTCTGCCTCCCTAGTGCT
P3	CGTCCAGAGGACCGAGGTT	AGTCTGTCATTCAGGCAATGG
P4	GGAAGTTCTTCAGAGAGCACG	GCCTGATGTCACTGCTTCCT
P5	CGGGACGACAGCTAAGGAAA	GTCTCGGGTTTGTTCACAGC
P6	AGCATCTTGCAGTTCCCACC	TGGAGCACCAAATGAGACTG
E1	CTTGCTGATCTCCCTAGCCC	AACTGCACAGGTGGGTGTTT
E2	GCACATTAACGCAGGCACTT	GGCTTGCCTGCAGACTATGG
E3	TGAATTGGGAGTGGTTCGAGG	GGTAGTGCTGTTTGGGGAGG

E4	GGTTAATGGGGAGAGCCAGC	CGGGAGACTGTTTGGGTCAG
